# Discovery of natural-product-derived sequanamycins as potent oral anti-tuberculosis agents

**DOI:** 10.1016/j.cell.2023.01.043

**Published:** 2023-03-02

**Authors:** Jidong Zhang, Christine Lair, Christine Roubert, Kwame Amaning, María Belén Barrio, Yannick Benedetti, Zhicheng Cui, Zhongliang Xing, Xiaojun Li, Scott G. Franzblau, Nicolas Baurin, Florence Bordon-Pallier, Cathy Cantalloube, Stephanie Sans, Sandra Silve, Isabelle Blanc, Laurent Fraisse, Alexey Rak, Lasse B. Jenner, Gulnara Yusupova, Marat Yusupov, Junjie Zhang, Takushi Kaneko, T.J. Yang, Nader Fotouhi, Eric Nuermberger, Sandeep Tyagi, Fabrice Betoudji, Anna Upton, James C. Sacchettini, Sophie Lagrange

**Affiliations:** 1 Sanofi R&D, Integrated Drug Discovery, CRVA, 94403 Vitry-sur-Seine, France; 2 Evotec ID (LYON) SAS, Lyon, France; 3 IGMBC, Strasbourg, France; 4 Global Alliance for TB Drug Development, New York, NY, USA; 5 Center for Tuberculosis Research, Johns Hopkins University School of Medicine, Baltimore, MD, USA; 6 Department of Biochemistry and Biophysics, Texas A&M University, College Station, TX, USA; 7 Institute for Tuberculosis Research, College of Pharmacy, University of Illinois at Chicago, Chicago, IL 60612, USA; 8 Sanofi R&D, DMPK, CRVA, 94403 Vitry-sur-Seine, France; 9 Sanofi R&D, Infectious Diseases TSU, 31036 Toulouse, France

**Keywords:** tuberculosis, antibacterial, macrolide, ribosome, emr37, drug discovery, sequanamycin, erythromycin, clarithromycin

## Abstract

The emergence of drug-resistant tuberculosis has created an urgent need for new anti-tubercular agents. Here, we report the discovery of a series of macrolides called sequanamycins with outstanding *in vitro* and *in vivo* activity against *Mycobacterium tuberculosis* (Mtb). Sequanamycins are bacterial ribosome inhibitors that interact with the ribosome in a similar manner to classic macrolides like erythromycin and clarithromycin, but with binding characteristics that allow them to overcome the inherent macrolide resistance of Mtb. Structures of the ribosome with bound inhibitors were used to optimize sequanamycin to produce the advanced lead compound SEQ-9. SEQ-9 was efficacious in mouse models of acute and chronic TB as a single agent, and it demonstrated bactericidal activity in a murine TB infection model in combination with other TB drugs. These results support further investigation of this series as TB clinical candidates, with the potential for use in new regimens against drug-susceptible and drug-resistant TB.

## Highlights

• Structure-based medicinal chemistry leads to an optimized sequanamycin-derived lead molecule, SEQ-9
• Sequanamycins are macrolide antibiotics able to overcome inherent Mtb macrolide resistance
• SEQ-9 is able to adjust its binding mode to the methylated (resistant) form of the Mtb ribosome
• SEQ-9 kills Mtb *in vitro* and is efficacious in mouse models of TB

## Graphical abstract

**Figure:**
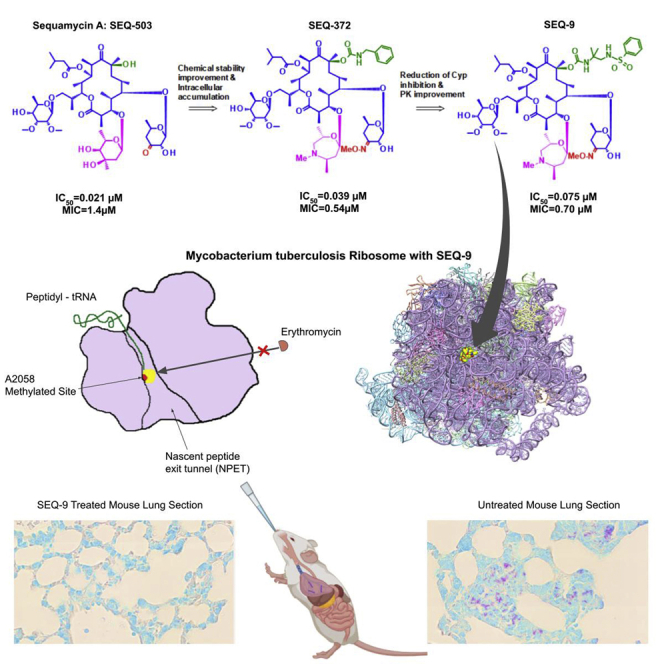

## Teaser

Macrolides generally have low efficacy against tuberculosis, but a class of macrolides called “sequanamycins” overcomes the resistance of *Mycobacterium tuberculosis* to macrolides and can be optimized for potent activity against a variety of *Mycobacterium tuberculosis* strains, including drug-resistant ones.

## Introduction

Tuberculosis (TB) is a respiratory infectious disease caused by the bacterium *Mycobacterium tuberculosis* (Mtb). Prior to the COVID-19 pandemic, TB ranked as the leading cause of infectious disease deaths worldwide, killing 1.45 million people and infecting 10 million more.^1^ Due to the increasing burden of multidrug-resistant TB (MDR-TB) and extensively drug-resistant TB (XDR-TB), there is a pressing need for novel TB drug combinations with activity against Mtb that is resistant to existing drugs. An ideal new regimen would combine universal activity with sufficient efficacy to cure patients in a shorter period than the current 6- to 9-month treatment course. It would be well-tolerated, require little to no monitoring for safety, and would be administrable orally. Several TB treatment regimens for both drug-susceptible and drug-resistant TB combining new or repurposed anti-TB drugs are being tested in late phase II or phase III trials. However, only three novel drug classes with little pre-existing resistance are included in these regimens—i.e., nitroimidazoles (delamanid or pretomanid), diarylquinolines (bedaquiline), and oxazolidinones (linezolid and sutezolid)—although there are several new compounds under development.^2^

### Antibacterial agents that target the TB ribosome

Linezolid is an antibiotic that inhibits protein synthesis by targeting the 50S ribosome. The toxicity of linezolid has so far prevented its general use against TB, but in a recent Korean clinical trial, it demonstrated a clear impact against XDR-TB.^3^ In addition, several late-phase trials evaluated the utility of linezolid against drug-resistant TB. Recently, the regimen bedaquiline-pretomanid-linezolid was approved by the US FDA for use against XDR-TB and treatment-intolerant MDR-TB, following the successful outcome of the Nix-TB trial that evaluated this all-oral regimen given for 6 months.^4^ However, linezolid toxicity was prevalent among patients treated in the Nix-TB trial, and the ongoing ZeNix-TB and TB-PRACTECAL trials aim to identify lower doses and/or durations of linezolid within bedaquiline-pretomanid-based regimens that contribute efficacy but are better tolerated by patients.^5^^,^^6^ In addition, data in preclinical models using linezolid and sutezolid demonstrated the potential of ribosome inhibitors to reduce the treatment duration needed for cure when given in combination with bedaquiline, pretomanid, and pyrazinamide.^7^^,^^8^^,^^9^

**Figure 1:**
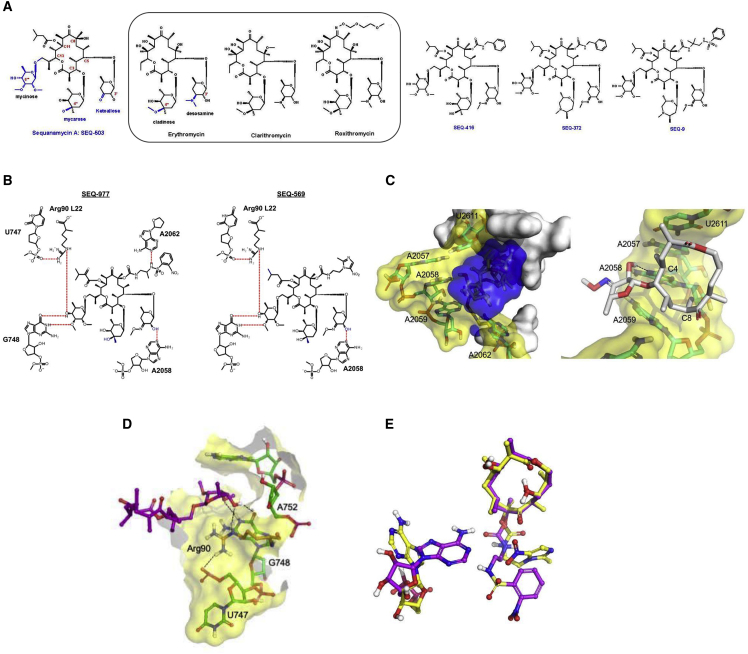
Key interactions of sequanamycins with the *T. thermophilus* ribosome For simplicity, only the lactone ring and the relevant side-chain substituents are shown in the 3D representations. Dotted black lines denote hydrogen bonds in the 3D picture; in the 2D interaction map, they are in red. The nucleotides that are the site of resistance mutations are labeled in red. (A) Structures of erythromycin and sequanamycin A and their derivatives. (B) The 2D interaction diagram of SEQ-977 and SEQ-569. (C) The fit of SEQ-977 (blue surface) and interactions formed between the wall of the peptide exit tunnel (yellow and white). The C5 ketoallose forms a hydrogen bond with A2058, with the hydrophobic C4-C8 edge of the central lactone making contact with the peptide wall, in an analogous manner to erythromycin. (D) The interactions formed by the mycinose at C13 of SEQ-977 and tunnel wall elements of L22 and domain II of 23S rRNA. (E) The relative position of A2062 in the presence of the carbamate at C8 for SEQ-569 (yellow) and SEQ-977 (purple).

Macrolides are another class of bacterial ribosome inhibitors that stop protein biosynthesis by blocking the polypeptide exit tunnel of the ribosome. Although this class of antibiotics are cornerstone drugs against many bacterial infections, they have been proven to be ineffective against TB. This is because macrolide antibiotics induce expression of the Mtb *erm37* gene that encodes a methyltransferase that methylates the Mtb ribosome^10^ primarily at position A2058 (unless otherwise stated, *E. coli* residue numbering is used throughout the paper, including the supplemental information). Although clarithromycin is occasionally used in salvage regimens for highly drug-resistant TB, it has never demonstrated significant efficacy against TB in the clinical setting or in preclinical models, and chemical optimization of the 14-membered ring macrolides has not met with success.^11^ Here, we describe the structure-guided optimization of the natural product SEQ-503 to the advanced lead SEQ-9 (Figure 1), which demonstrates *in vitro* activity and potent *in vivo* efficacy in mouse models of acute and chronic TB.

## Results

### Sequanamycins are active against TB methylated ribosome

Discovered in 1969, sequanamycin A (SEQ-503) is a 14-membered ring erythromycin family macrolide produced by *Allokutzneria albata*, a member of the family *Pseudonocardiaceae* in the order *Actinomycetales*. SEQ-503 exhibits a good minimum inhibitory concentration (MIC) of 1.4 μM for Mtb, while most macrolide antibiotics have much higher MICs (erythromycin 128 μM, clarithromycin 8 μM, and roxithromycin 32 μM). Furthermore, SEQ-503 differs structurally from other 14-membered macrolides by having mycarose at C3 instead of cladinose, a ketoallose at C5 instead of desosamine, and a mycinose at position C13 (Figure 1A). Members of the sequanamycin family bind in the same pocket of the ribosome as erythromycin (Figures S2 and S3).

This increased potency demonstrated the potential of SEQ-503 as an anti-tubercular agent, but this compound presented several challenges. SEQ-503 was unstable in acidic media with a measured half-life of less than 30 min at pH 2 in 50% acetonitrile/water, suggesting it would undergo rapid degradation in the human stomach, and was thus not a good candidate for an oral drug. Furthermore, the compound was metabolically unstable in human liver microsomes, with 67% of compound metabolized *in vitro* within 20 min (Table 1). A medicinal chemistry effort was undertaken aiming to overcome these limitations. Two sequanamycin derivatives, SEQ-569 and SEQ-977, discovered through serial synthetic modifications of SEQ-503, showed improved potency against the Mtb H37Rv strain (MIC: 0.57 and 0.13 μM, respectively). These two derivatives differ in the substituents found in the carbamate group at C8.

**Table 1:** *In vitro* activity, chemical and ADME parameters, and PK parameters, for SEQ-9 and analogs

	SEQ-503	SEQ-416	SEQ-372	SEQ-9
Inhibition of Mtb translation (IC_50_ μM)	0.021	0.044	0.039	0.075
Translation inhibition of Mtb methylated ribosome (IC_50_ μM)	0.022	0.031	0.054	0.064
MIC for Mtb in liquid culture (μM)	1.4	0.15	0.54	0.70
MIC for Mtb tuberculosis in macrophages (μM)	2.5	0.63	0.06	0.11
Metabolic stability in human liver microsomes (% metabolized compound after 20 min)	67	40	33	10
	4(K)	0.5(K)	19(K)	0(K)
	/41(Q)	/35(Q)	/32 (Q)	/10(Q)
Metabolic stability in mouse/rat liver microsomes (% metabolized compound after 20 min)	0/ND	25/22	8/22	8/9
Metabolic degradation/clearance from human liver microsomes (mL/h/10^6^ hepatocytes)	ND	0.057	0.006	0.03
CYP3A4 inhibition (μM)	>10	3(M)/10(T)	1.2(M)/3.8(T)	>30(M)/>50(T)
Chemical stability: t/12@pH 2	<30 min	∼6 h	∼25 h	48 h
Solubility: PBS@pH 7.4 (μg/mL)	522	22	58.8	51
Solubility: MCT@pH 7.4 (μg/mL)	ND	599	1,420	530
LogD (pH 7.4)	ND	4.5	4.93	3.4
pK_a_	–	–	8.3	8.3
Permeability in Caco2 cells (Papp: 10^−7^ cm/s)	0.7	104	22	15
Oral bioavailability (F) (%) mouse	22%	21%	17%	23%
AUC (ng·h/mL) in plasma @30 mg/kg	ND	4,000	900	6,300
AUC (ng·h/mL) in lung @30 mg/kg	ND	4,800	18,000	120,000

Parameters for sequanamycin derivatives: metabolic stability in % after 20 min incubation in presence of ketoconazole CYP3A inhibitor (K), metabolic stability in % after 20 min incubation in presence of quinidine CYP2D6 inhibitor; CYP3A4 reversible inhibition: IC_50_ determination with midazolam as substrate (M), IC_50_ determination with testosterone as substrate (T) AUC, area under the curve.

**Figure S1:**
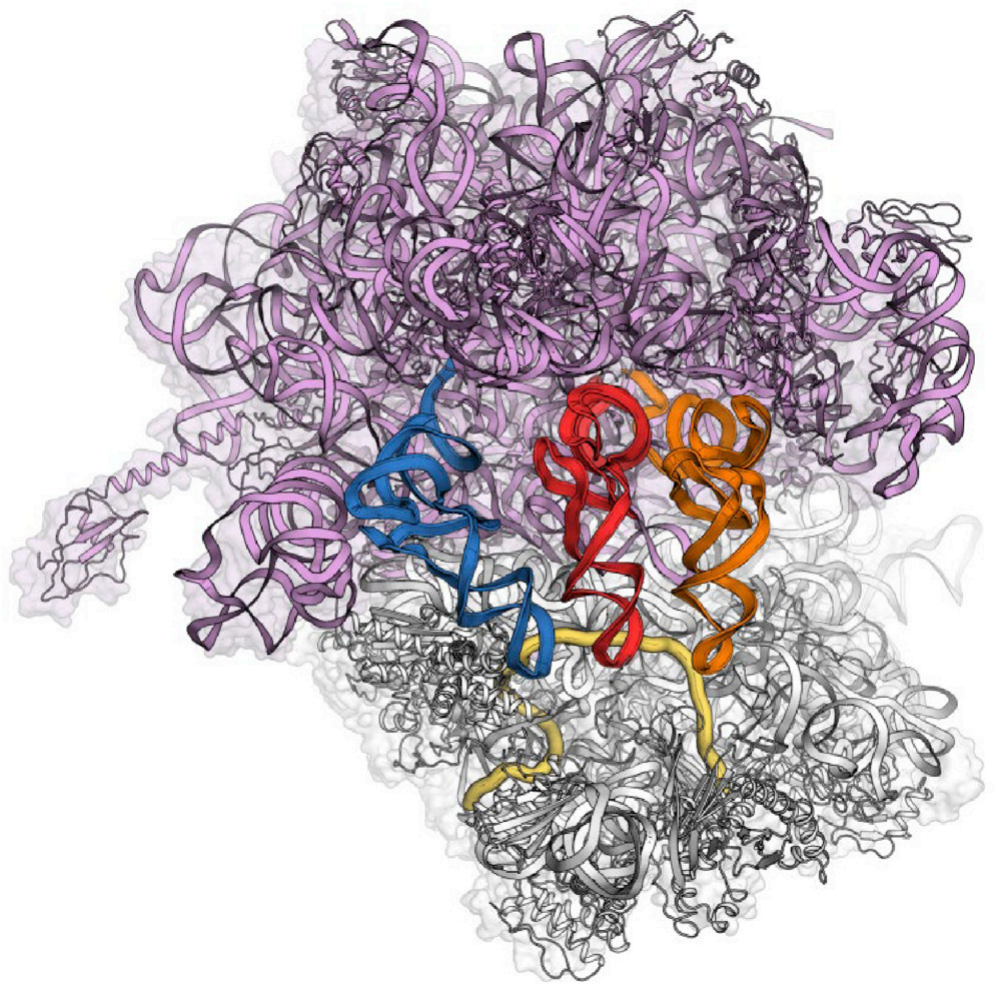
*T. thermophilus* 70S ribosome in complex with tRNA^phe^ (in all three tRNA binding sites—E in blue, P in red, and A in orange) and mRNA (yellow), related to Figure 1

**Figure S2:**
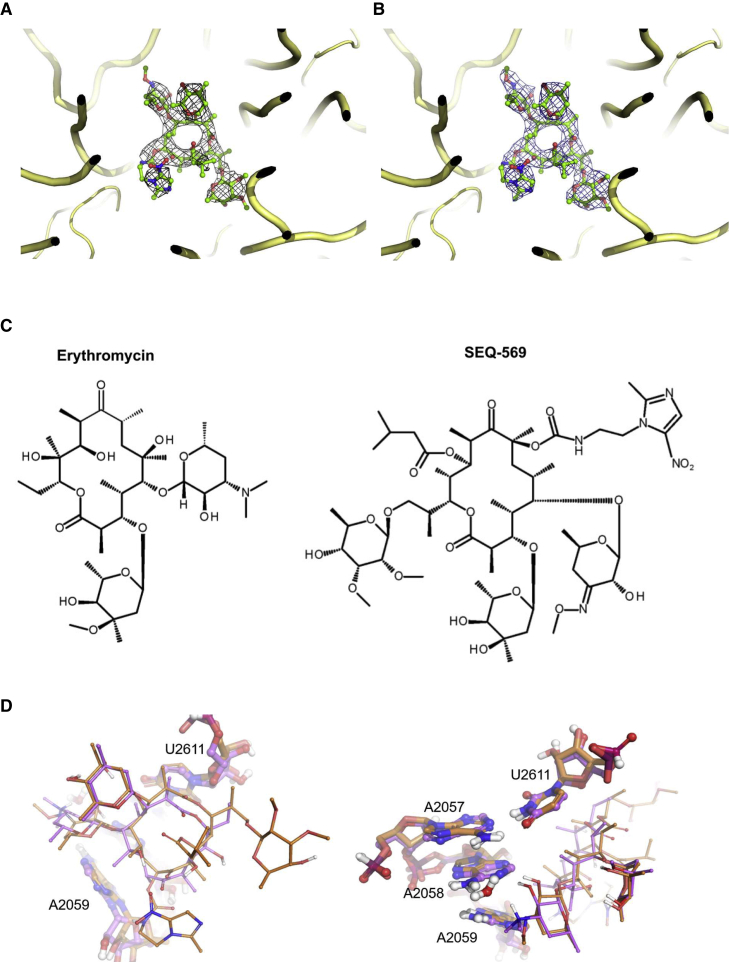
Structures and electron density of SEQ-569 complexed to *T. thermophilus* ribosome, related to Figures 1 and 2 (A) Unbiased F_o_-F_c_ electron density map of the *T. thermophilus* structure with SEQ-569 contoured at 3σ. (B) Final 2F_o_-F_c_ electron density around SEQ-569 contoured at 1σ. (C) The 2D chemical structures of erythromycin and SEQ-569. (D) Views of SEQ-569 (brown) aligned to erythromycin (violet, PDB: 6XHX) show the two antibiotics are similarly positioned in the ribosome, with the stabilizing lactone ring methyl groups equivalently positioned.

**Figure S3:**
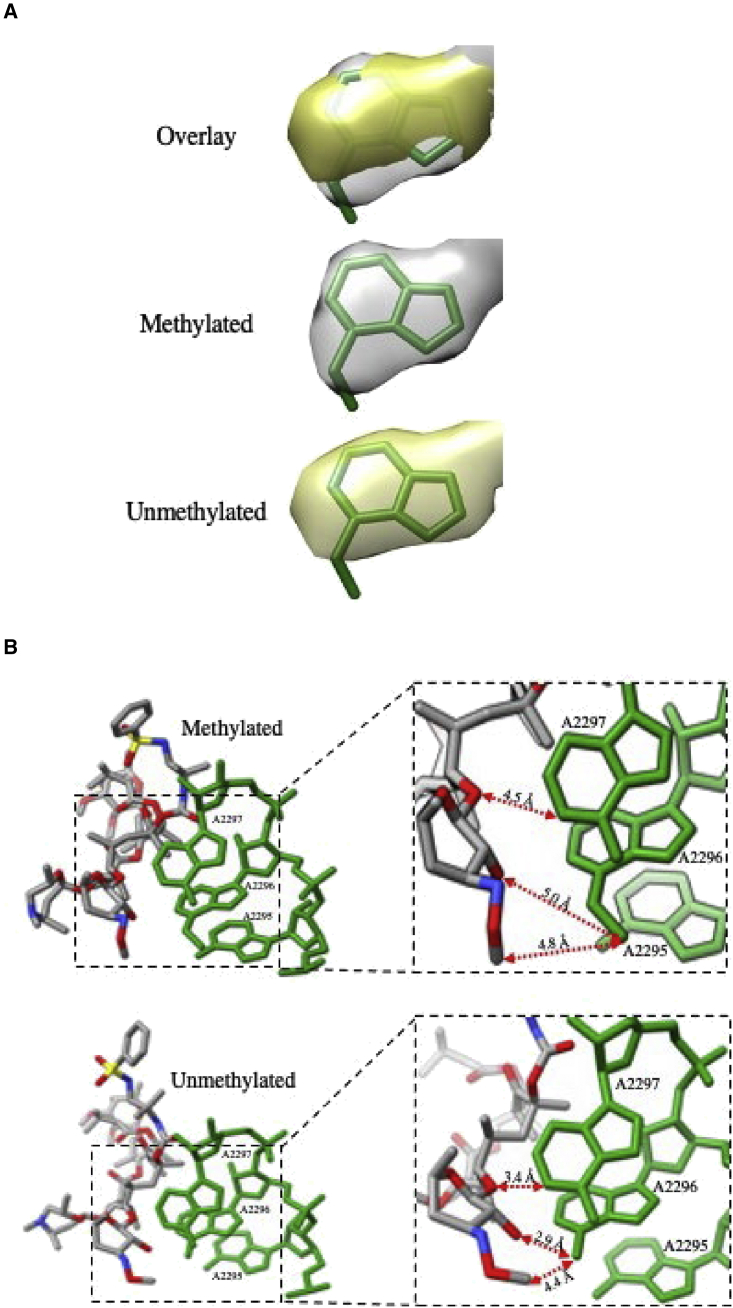
Comparisons of cryo-EM structures of methylated and unmethylated Mtb ribosome, related to Figure 3 (A) Cryo-EM density for the methylated and unmethylated base A2058. SEQ-9 is colored by element, and nucleotides are colored green. (B) There is additional density observed for the methylated N-6 position of A2058 (A2296 Mtb numbering) and (B) methylation-based conformational changes observed in the structures of SEQ-9 and nucleotides including close-up views of the boxed regions in the two ribosome models showing movements of several functional groups in response to the methylation of the ribosome.

The crystal structure of the *Thermus thermophilus* (*T. thermophilus*) ribosome was used as a model^12^^,^^13^ to guide the optimization of the sequanamycins. We succeeded in co-crystallizing SEQ-569 and SEQ-977 bound to the *T. thermophilus* 70S ribosomes (Figure 1). The structures of the *T. thermophilus* 70S ribosome with bound SEQ-569 (PDB: 7AZS) and SEQ-977 (PDB: 7AZO) were determined to 3.2 and 3.4 Å resolutions, respectively (Tables S1 and S2). The structures contained tRNA^Phe^ in all three tRNA binding sites and mRNA specifically constructed to mimic the state of the ribosome after several rounds of translation (Figure S1). Overall, the structures were similar to that of the *T. thermophilus* ribosome with erythromycin,^13^ and the active sites displayed clear electron density for sequanamycin in the macrolide binding pocket (Figures S2 and S3). Like other macrolides, the sequanamycins bind in a narrow part of the peptide exit tunnel at a site that lies between the peptidyl transferase center and a constriction in the tunnel that occludes the lumen, near ribosomal proteins L4 and L22. Their lactone rings bind to the peptide tunnel with the hydrophobic faces of their lactone rings facing the wall of the exit tunnel forming van der Waals contacts, while the hydrophilic faces are exposed to solvent. Specifically, the C4–C8 edge of the lactone ring contacts the hydrophobic surface formed between A2057 and A2059, and the lactone ring side chains apparently contribute significantly to the binding. We observed a hydrogen bond between the 3′-hydroxyl of the ketoallose group at C5 and N1 on A2058. Moreover, the carbamate group linked to C8 of the lactone ring and an additional sugar mycinose attached to C13 extend the long axis of the molecule down the exit tunnel, away from the peptidyl transferase center. The mycinose group hydrogen bonds with Arg92 of the L22. In addition, G748 forms a hydrogen bond with the 5′′′-methoxy group that sits in a cavity formed by G748 and A752. Compared with classical erythromycin macrolides, we hypothesize that these additional hydrogen bonds are critical for sequanamycins as a second anchoring position to the ribosome. In the ribosome crystal structure with SEQ-569, adenine A2062 flips about 90° from its position facing into the lumen of the peptide tunnel to a position that opens the peptide tunnel in response to antibiotic binding.

The overall structure of SEQ-977 bound to the ribosome is similar to that of SEQ-569 except for the carbamate group at C8, on which the NH of the sulfonylamide group forms a hydrogen bond with N1 of A2062. This interaction causes A2062 to orient itself differently in the SEQ-977 co-crystal structure, compared with SEQ-569 (Figure 1). The additional hydrogen bond may explain the increased potency of SEQ-977 (IC_50_ = 0.034 μM, MIC = 0.13 μM) over SEQ-569 (IC_50_ = 0.054 μM, MIC = 0.57 μM).

### SEQ-9 shows potent, on-target activity against the Mtb ribosome

**Figure S4:**
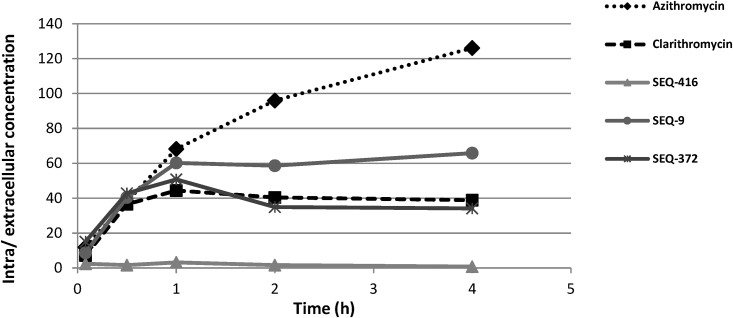
Intra-macrophage uptake of sequanamycin derivatives expressed as the ratio of the intra versus extracellular concentration, related to STAR Methods section intra-macrophage assay EQ-372 and SEQ-9’s improved activities inside macrophages, compared with their neutral counterpart SEQ-416, is due to higher concentration in macrophage cells. RAW macrophages were exposed to SEQ-372 and SEQ-9 and SEQ-416 at 10 μM. After lysis at different time points, the compound’s cellular concentrations were measured by LC-MS/MS. To compare the potency to accumulate in cells of each compound, intra/extra cellular concentration ratios were calculated. Clarithromycin and azithromycin were used as references. Clarithromycin showed saturable intra-macrophage accumulation, while azithromycin gave an unsaturable accumulation due to its bi-basic character. SEQ-372 showed similar intra-macrophage profile to clarithromycin, while SEQ-9 demonstrated a higher intra-macrophage ratio (∼60-fold).

**Figure S5:**
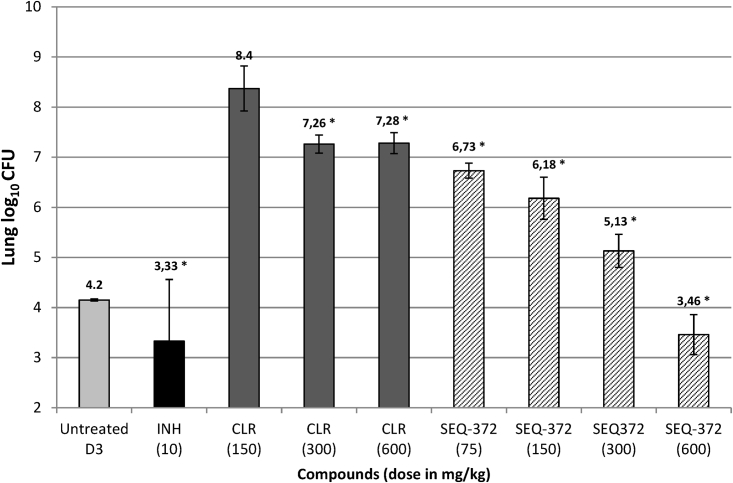
SEQ-372 efficacy in mouse model of acute tuberculosis, related to Figure 5 Bacterial loads (CFU) were enumerated in the lungs of mice treated with the isoniazid (INH), clarithromycin (CLR), and SEQ-372 for 1 month and sacrificed at day 31 after infection. Histograms represent the mean lung log_10_ CFU counts (5 mice per group) after 4 weeks of treatment. A group of five mice were sacrificed 3 days after infection to measure lung CFU burden at treatment initiation. Vehicle control mice died prior to day 31.

Further optimization was centered on modifying the ketoallose at C5 and the carbamate group at C8 and resulted in compounds, such as SEQ-416, with improved potency against Mtb and chemical stability but with modest efficacy in murine TB models. We hypothesized that these results were related to limited penetration into and accumulation in macrophages (Figure S4) where most Mtb reside under pathological conditions.^14^ Further modification of the mycarose into a 7-membered 1,4-oxazepane ring with a substituted amine gave rise to SEQ-372, with weak basicity (pK_a_ of 8.2) and increased lipophilicity (logD of 4.93), physiochemical properties known to improve intracellular compound accumulation.^15^^,^^16^ Compared with its neutral analog SEQ-416 (IC_50_ = 0.044 μM, MIC = 0.15 μM), SEQ-372 (IC_50_ = 0.039 μM, MIC = 0.54 μM) was slightly less potent against growing bacteria under standard MIC assay conditions but exhibited a 20-fold increase in macrophage accumulation (Figure S4) and approximately 10-fold increased potency against Mtb in the macrophage (Table 1). SEQ-372 was the first compound in the series to display dose-dependent efficacy in a murine model of acute TB infection, being more potent than clarithromycin at the same dose in the same assay (Figure S5). However, SEQ-372 inhibited cytochrome 3A4, which is not a desirable property for a drug used in a combination therapy. Further optimization of the carbamate moiety at C8 resulted in SEQ-9 (IC_50_ = 0.065 μM), which demonstrated desirable absorption, distribution, metabolism, and elimination (ADME) properties. The half-life of the compound under acidic conditions (pH 2.0), in 50% acetonitrile/water, increased from 30 min for SEQ-503 to 48 h. Metabolic liabilities were found to be lower in human, mouse, and rat microsomes (Table 1), resulting in a 6-fold higher plasma area under the curve (AUC) of SEQ-9, compared with SEQ-372 administered at the same oral dose of 30 mg/kg in mouse. Moreover, the lung over plasma exposure ratio was 19, indicating exceptional distribution into lung tissue. Cytochrome 3A4 inhibition activity was also abolished in SEQ-9. We also found that the *in vitro* cytotoxicity in HepG2 cells (IC_50_ = 10 μM) and primary human hepatocytes (IC_50_ = 26 μM) suggested that SEQ-9 had a reasonable *in vitro* safety margin, compared with its MIC against Mtb. We tested this analog for activity against methylated ribosomes. A Mtb culture treated with erythromycin at 1 μg/mL for 24 h was used to induce the expression of *erm*37, and the purified ribosome was then added into cell-free extracts (S100) for the measurement of macrolide protein synthesis inhibition dose responses. SEQ-9 maintained its potency in the cell-free protein synthesis assay, despite the addition of methylated ribosomes, whereas a 100-fold increase in the IC_50_ values was observed in this assay for erythromycin, clarithromycin, azithromycin, tylosin, and clindamycin (Table S3).

*Mycobacterium bovis* (*M. bovis*) BCG Pasteur strain (RD2 deletion vaccine strain) was used to check the effect of *erm*37 expression on sequanamycin resistance. Unlike Mtb, this strain is sensitive to clarithromycin with an MIC of 0.1 versus 2 μM in Mtb because it lacks a functional Erm37 protein. The MICs for modified sequanamycins were similar in both Pasteur *M. bovis* BCG and Mtb (SEQ-372: 0.28 versus 0.54 μM and SEQ-9: 0.32 versus 0.7 μM, respectively). By cloning *erm37* under the control of an inducible promoter, we showed that *erm37* expression significantly increased the MICs of clarithromycin and erythromycin, but the activities of SEQ-372 and SEQ-9 were unaffected. Furthermore, pre-incubation of Mtb with subinhibitory concentrations of SEQ-9 induced *erm37* mRNA expression (Table S5). Interestingly, pre-incubation did not affect SEQ-9 potency but induced resistance to clarithromycin (Tables S4 and S5). These findings demonstrate that despite the difference in structural features at the C3 position (a 7-membered 1,4-oxazepane ring in SEQ-9 versus a cladinose in erythromycin), sequanamycins, like erythromycin, triggers the expression of Mtb *erm37* that has been linked to either programmed ribosome stalling^17^ or induction of WhiB7.^18^ However, unlike other macrolides, sequanamycins avoid the self-induced resistance in Mtb mediated by the expression of *erm37*.

**Figure 2:**
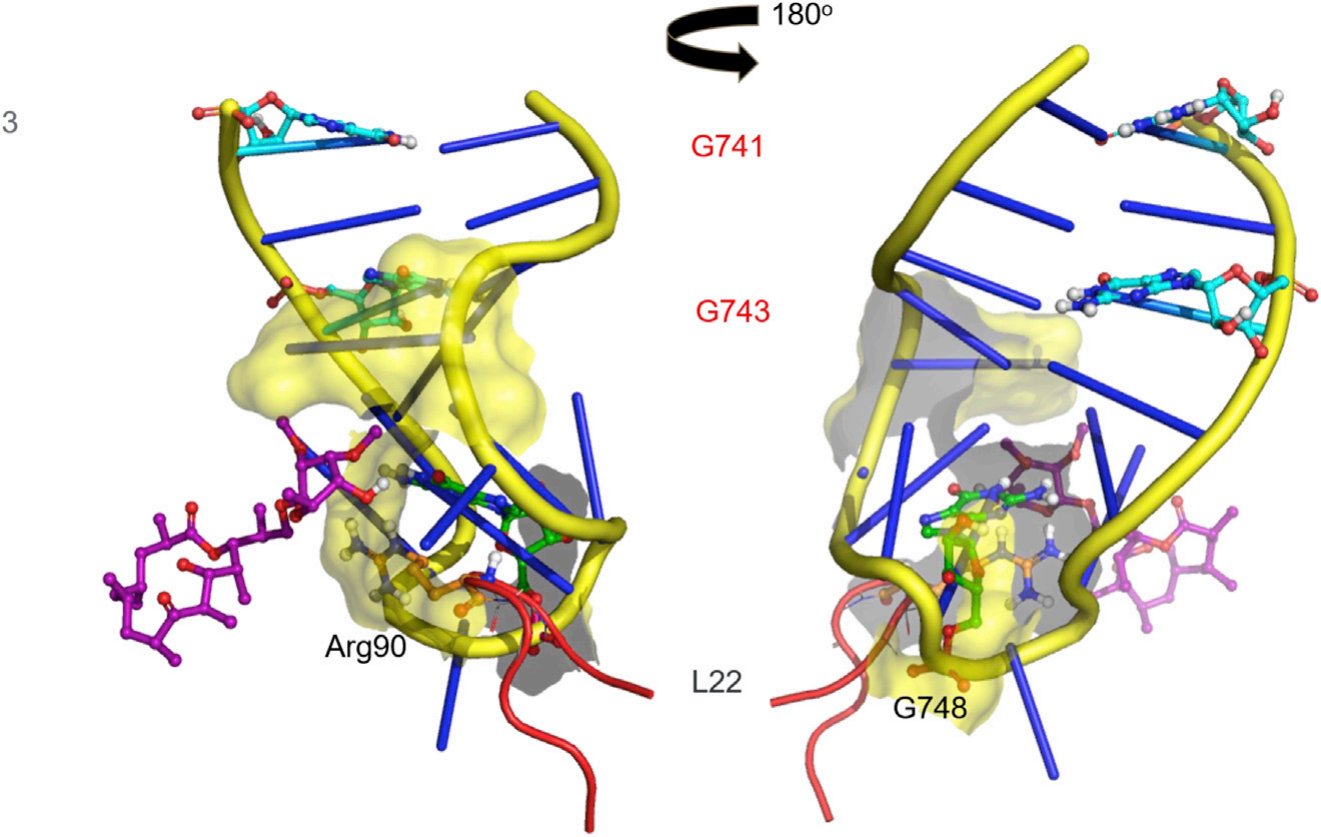
The position of G741 and G743 (light blue—appears green with yellow overlay on the left image) in helix 35 adjacent to the C13 mycinose of SEQ-977 Deletion of G741 and G743 would alter the conformation of the helix, disrupting the interactions by the mycinose group with the helix and the L22 Arg90.

To evaluate the frequency of resistance to SEQ-9, and to confirm on-target activity, we isolated spontaneous Mtb mutants resistant to SEQ-9. The frequency of resistant mutations to SEQ-9 was approximately 10^−8^ against the H37Rv strain of Mtb, determined at 4 × MIC. This is comparable to that of several TB drugs (isoniazid, ethambutol, pretomanid, and delamanid) and in the range of others (rifampicin, bedaquiline, and linezolid).^19^^,^^20^^,^^21^^,^^22^ Resistant mutants selected with SEQ-9 were found to have higher resistance to sequanamycin antibiotics and to clarithromycin, while remaining sensitive to rifampicin and linezolid. We sequenced the genomes of the mutants and found mutations in the 23S rRNA: one within domain V (A2058G), a deletion of a guanine at position 1659, and two deletions in helix 35 of domain II (G741 and G743), in a region that interacts with the L22 protein (Table S6). The deletion of bases G741 and G743 in *T. thermophilus* (equivalent to G872 and T874 in Mtb; Table S6) abolished SEQ-9 activity. They are situated a few bases away from the key mycinose-anchoring elements, in close proximity to the mycinose-anchoring residues at the base of helix 35 (Figure 2). These mutations are likely to result in significant structural rearrangement and positioning of the terminal hairpin loop, possibly resulting in the partial occlusion of the peptide exiting tunnel. These changes would account for the loss of binding of the sequanamycins and interestingly, the loss of clarithromycin activity, which lacks a mycinose group.

Helix 35 is of critical importance to sequanamycin binding. G748, A750, and A752, of helix 35, form a pocket that binds the mycinose group of SEQ-9. We found that U747 and G748, also located at the base of the helix, play a key role in orienting the guanidinium group of Arg90 in L22—a key residue that binds the mycinose moiety of the sequanamycins (Figure 1D).

The resistant mutant, where A2058 was mutated to guanine, further defines the important binding interactions of the sequanamycins (Table S6). According to previous published results, the A to G mutation resulted in a steric clash between the C2 amine of G2058 and the C6 methyl group of the lactone ring. The forced displacement of the lactone ring abolishes binding, explaining the lack of activity of the sequanamycins on G2058 ribosomes, as genome sequencing and structural data reveal that in the human mitochondria, this position is a guanine.^23^

### Cryo-EM structure of the Mtb ribosome bound to SEQ-9

**Figure 3:**
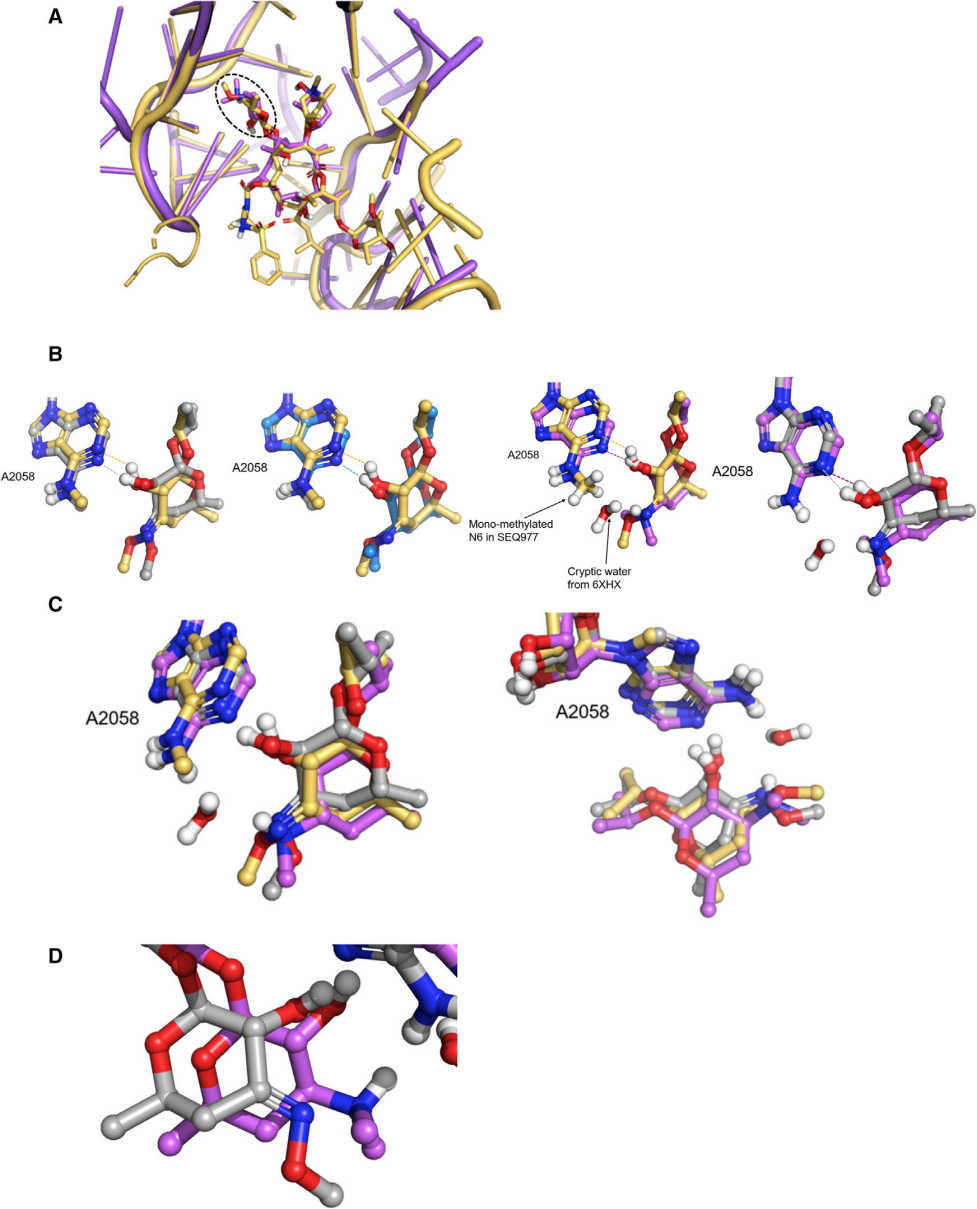
The positioning and interactions of the ketoallose at C5 with A2058 Alignment was achieved by superposing the 23S rRNA elements. (A) The position of SEQ-977 (gray) and erythromycin (violet, PDB: 6XHX and blue, PDB: 4V7X) in *T. thermophilus*, along with the cryo-EM structure of SEQ-9 (yellow) bound to the Mtb ribosome. The hashed black line denotes the position of the ketoallose group in the SEQ-977 structure, which is also positioned equivalently in the other structures. (B) The hydrogen bonds formed between the 3′-hydroxyl group of the respective macrolides and the N1 group of A2058. The cryptic water and the hydrogen bond it forms with the N6 group of A2058 and the dimethylamine group of erythromycin are also shown. (C) Overlay and different views of the ketoallose groups of the three macrolides and their respective A2058 residues. The different views illustrate the slight difference in orientation of the methylated A2058 residue in the Mtb-SEQ-9 cryo-EM structure, compared with the *T. thermophilus* structures. (D) The charged sp3 hydribized dimethylamine of erythromycin (6XHX) compared with the neutral sp2 hydribized oxime of SEQ-977.

Although the binding sites of the *T. thermophilus* and Mtb ribosome are similar (Figure 3A), in order to better understand the mechanism by which sequanamycins overcome resistance from the methylation of A2058 on the Mtb ribosome, we performed cryo-EM studies to determine the structure of the A2058-methylated Mtb ribosome with SEQ-9 bound (PDB: 7KGB, EMDB: EMD-22865) and, for comparison, the structure of the unmethylated Mtb ribosome bound to SEQ-9 (PDB: 7SFR, EMDB: EMD-25100).

Methylation of the ribosome was accomplished by growing Mtb with subinhibitory concentrations of erythromycin in the culture media, and methylation of the ribosome was confirmed by measuring the shift in the IC_50_ of the methylated ribisome compared with the unmethylated ribosome. The unmethylated ribosome had an IC_50_ for erythromycin and clarithromycin about 200× lower, compared with the ribosome produced in the presence of subinhibitory erythromycin, in agreement with what has been observed by Madsen et al.^24^ The cryo-EM structure shows density for a methyl group on the N6 position of A2058; however, there was no extra density indicating methylation for A2057 or A2059.^25^ In addition to the extra density, there was a significant movement of SEQ-9 away from A2058 in the methylated ribosome as compared with the unmethylated ribosome (Figure S3). The distance from the N6 of A2058 to the 3′-hydroxyl oxygen of SEQ-9 is 2.9 Å in the unmethylated ribosome and 3.7 Å in the methylated ribosome. The distance from the N1 of A2059 to the oxygen linking the ketoallose group to the C5 position of the lactone ring shifts from 3.4 Å in the unmethylated ribosome to 4.5 Å in the methylated ribosome.

**Figure S6:**
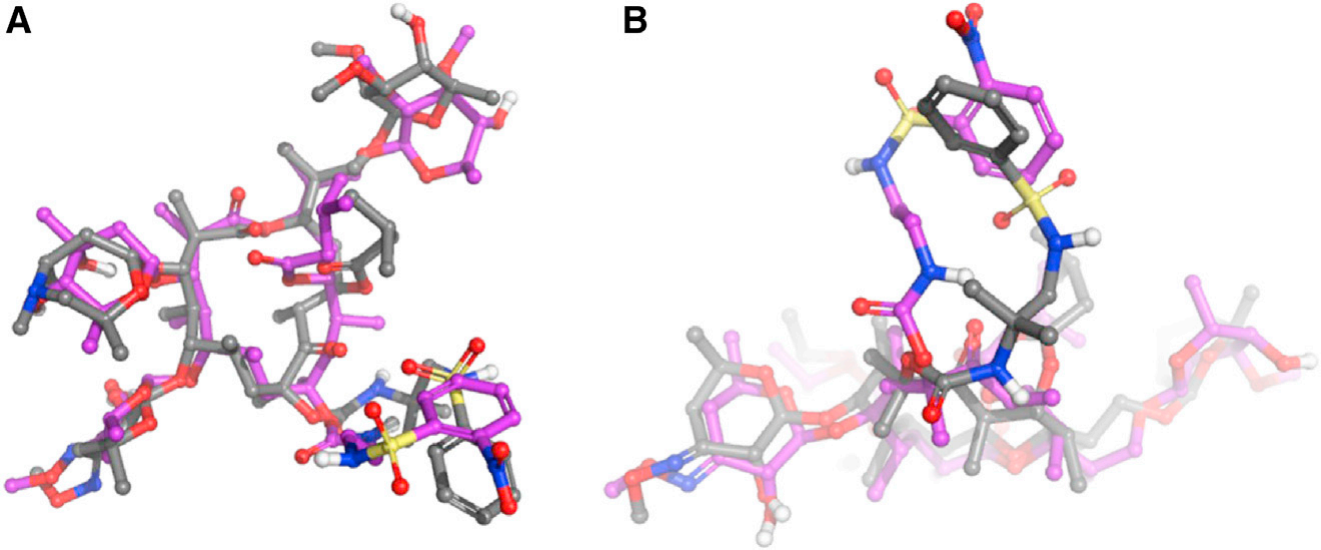
A comparison of the macrolides structures in the X-ray of *T. thermophilus* and cryo-EM of methylated Mtb, related to Figures 1 and 2 (A) The overlay of SEQ-977 (purple) and SEQ-9 (gray) shows the relative position of the macrolide side chains to be similar. (B) In spite of the similar relative positioning of the C8 side chains, SEQ-977 forms a hydrogen bond with A2062, unlike SEQ-9.

For the methylated Mtb ribosome, SEQ-9 still showed potent inhibition of protein synthesis (IC_50_ = 0.075 and 0.065 μM for the methylated Mtb ribosome), which suggests the methyl group on N6 of A2058 does not reduce the overall binding interactions between the Mtb ribosome and sequanamycins. Despite the presence of additional helical stem-loops in the 23S rRNA, a comparison of the crystal structure of the *T. thermophilus* ribosome with that of the cryo-EM methylated ribosome (Figure S6) indicates both macro-lactone and ketoallose groups are located in the same pockets. However, the 3′-hydroxyl is shifted, compared with the *T. thermophilus* structure, and the 4′-methyloxime of the ketoallose protrudes differently in methylated Mtb (Figure 3). A structure of the *Staphylococcus aureus* (*S. aureus*) ribosome suggested that macrolide resistance is caused by a steric clash between the methyl group of N6 on A2058 and the dimethyl amine group of the desosamine sugar.^23^ However, more recent crystal structures of erythromycin bound to *T. thermophilus*^26^ have raised questions about this conclusion and highlighted the role methylated A2058 plays in displacing a water molecule that is key to mediating antibiotic binding to the ribosome (Figures 3B and 3C). We superimposed the erythromycin structures^13^^,^^17^^,^^27^ (PDB: 4V7X, 4V7U, and 6XHX) with the SEQ-9-bound A2058-methylated Mtb ribosome structure, examining the interactions with the mono-methylated A2058 Mtb ribosome (Figure 3B). Despite the observed shift of the 3′-hydroxyl group, a hydrogen bond is observed between the hydroxyl group and the N1 of A2058. Because of the hydrogen bond, the shift of the hydroxyl group induces a movement of A2058 such that the methylated N6 nitrogen is at a slightly different angle to the SEQ-9, compared with its position when erythromycin is bound.

The repositioning of A2058, coupled with the fact that the sequanamycins lack the bulky dimethyl group adjacent to the N6, eliminates the possibility of a steric clash between the methylated N6 group on A2058 and sequanamycins. Furthermore, the water molecule that mediates a tripartate interaction between the desoamine moiety of erythromycin, the N6 amine group of A2058, and the backbone of G2505 is unlikely to form equivalent interactions with sequinamycin due to the sp2 hybridization of the oxime group, compared with the sp3 dimethylamine of erythromycin (Figure 3D). Moreover, the structural alignments clearly demonstrate methylated A2058 would displace the water molecule (Figure 3B).

**Figure 4:**
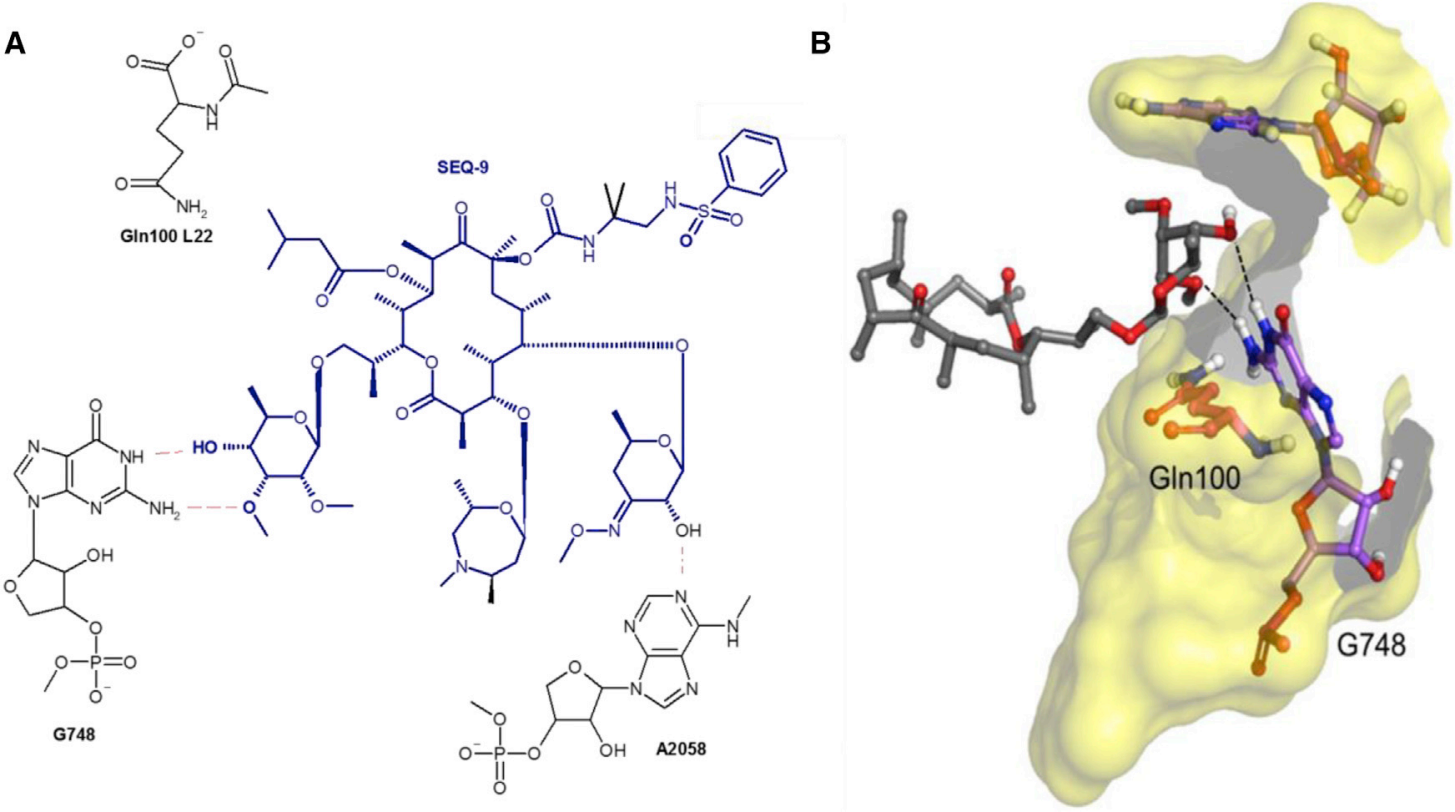
Key interactions of SEQ-9 with the Mtb ribosome Dotted black lines denote hydrogen bonds. (A) The 2D interaction diagram of SEQ-9 and (B) the bidentate interaction formed by the mycinose C13 and G748 of the Mtb ribosome, which differ from the interactions formed in *T. thermophilus*. Also, unlike *T. thermophilus*, the Mtb L22 Arg90 equivalent, Gln100, does not form a hydrogen bond with the mycinose of SEQ-9.

In contrast to the hydrogen bond between mycinose at C13 and Arg90 in L22 in the crystal of the *T. thermophilus* ribosome complex, the cryo-EM structure indicated that a double bidentate interaction is formed between the N6, NH2 at C7 of G748 and the hydroxyl group and methoxyl group of the mycinose sugar (Figure 4). Moreover, the additional hydrogen bond in SEQ-977, formed between the NH of the sulfonylamide from the carbamate group at C8 and N1 of A2062, is not observed here: A2062 lines up along the wall of the tunnel in an analogous manner to the SEQ-569 structure (Figure 1E). However, the weak electron density of the C13 and C8 side chains in the cryo-EM density map of the Mtb ribosome-SEQ-9 complex suggests that these side chains may be quite flexible and interact with the peptide tunnel wall through non-specific interactions. This has also been observed on ketolides that have an 11,12-linked carbamate and an extended alkyl-aryl side chain. The alkyl-aryl side chain stretches down the exit tunnel away from the peptidyl transferase center, protecting A752 in the *E. coli* and *S. aureus* ribosomes but not in the *D. radiodurans* or *H. halobium* ribosomes.^28^^,^^29^ The orientations of the ketolide pharmacophoric side chain varies when the inhibitor binds to ribosomes from different species.^11^

### SEQ-9 is active against drug-resistant and susceptible Mtb strains

**Figure S7:**
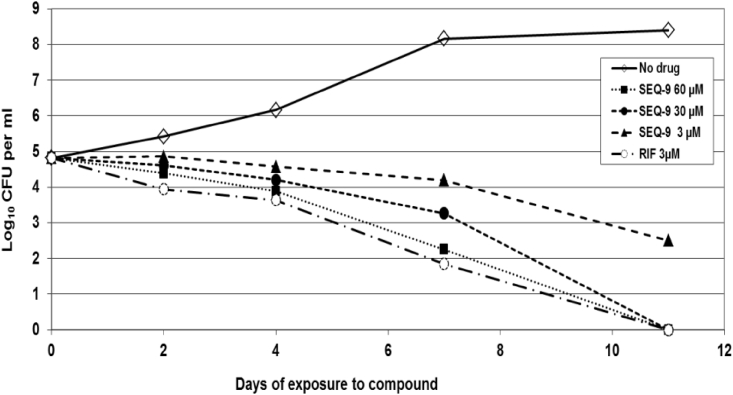
SEQ-9 kill kinetics of Mtb H37Rv in 7H9 broth culture, related to Figure 5 Bacterial cultures with various SEQ-9 concentrations or 1% DMSO were followed during 11 days. For each time, CFU count was assessed by plating on 7H11 agar plates. The number following the compound abbreviation indicates the concentration in μM.

SEQ-9 exhibited potent antibacterial activity against a variety of Mtb strains. The MIC values of SEQ-9 were similar for a diverse panel of Mtb clinical isolates and strains resistant to individual first- or second-line TB drugs (Table 2). SEQ-9 also exhibited activity against non-replicating Mtb under hypoxic conditions with an MIC of 0.6 μM. Time-dependent, *in vitro* bactericidal activity was also demonstrated by SEQ-9, with a 1.5 log colony-forming unit (CFU) reduction at 30 μM after a 7-day treatment and a 2.5 log CFU reduction at 60 μM over the same time frame (Figure S7).

**Table 2:** Activities of SEQ-9 against different Mtb strains and under different assay conditions

Assays	Strain	SEQ-9
MIC (μM) for Mtb in liquid culture	H37Rv/Erdman	0.7
MIC (μM) for Mtb in liquid culture + 25% human serum	H37Rv LuxAB	1.2
MIC (μM) for Mtb in liquid culture + 25% mouse serum	H37Rv LuxAB	2.1
MIC/minimal bactericidal concentration (MBC) (μM) for Mtb under anaerobic conditions (LORA, low-oxygen recovery assay)	H37Rv LuxAB	0.6/6.3
MIC (μM) for Mtb in macrophages	H37Rv GFP	0.15
MIC (μM) against drug-resistant and susceptible Mtb clinical strains	KMA1354-Modern KMA3899-Ancient KMA4244-Modern KMA4439-Modern KMA5282-Modern KMA5319-Modern	0.9 1.4 1.4 1.4 0.6 0.7
MIC (μM) against mono-drug-resistant Mtb	rINH rRIF rCAP rCYS rKAN rMOX	0.6 0.6 0.7 0.8 0.5 0.8

**Figure S8:**
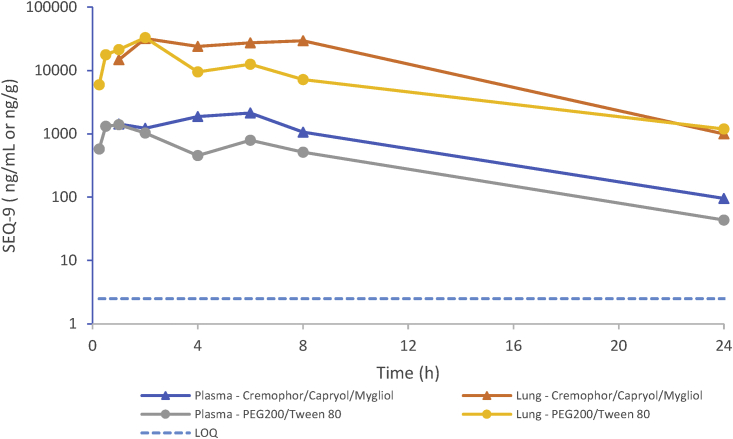
PK profile of SEQ-9 at 100 mg/kg, related to STAR Methods section pharmacokinetic studies in mice SEQ-9 concentration was determined in plasma and lung, using an exploratory LC-MS/MS assay method after single oral dose of SEQ-9 100 mg/kg administered either as a solution (PEG200/Tween 80) or as lipidic formulation (Cremophor/Capryol/Mygliol). Concentration in plasma corresponds to the mean of 3 individual results for each time point. Concentration in lung corresponds to the result of pooled samples (n = 3) for each time point.

### SEQ-9 is efficacious in mouse models of TB

**Figure 5:**
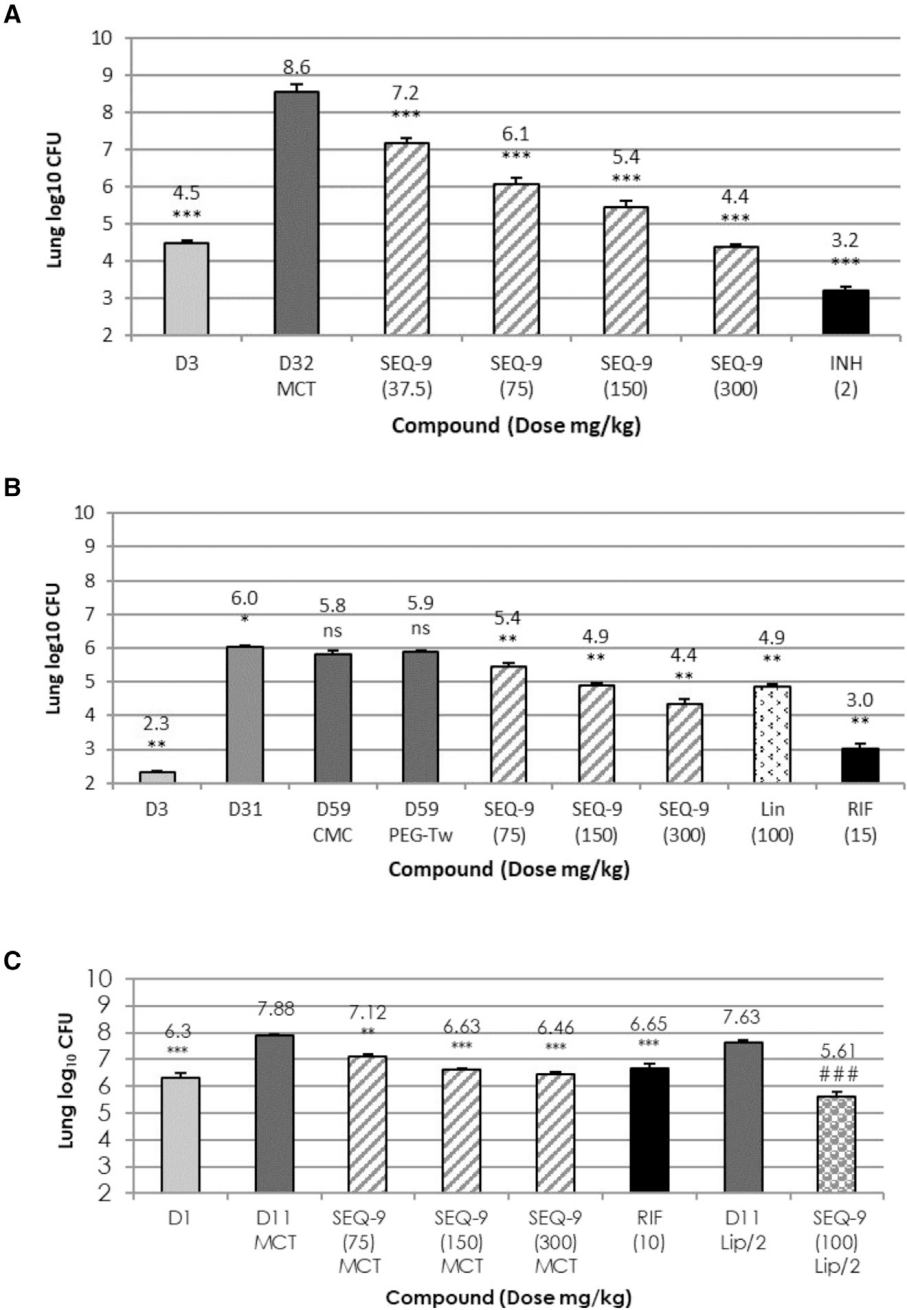
*In vivo* activity of SEQ-9 in the lungs of Mtb-infected BALB/c mice after treatment with SEQ-9 Histograms represent the mean lung log_10_ CFU counts (5–10 mice per group). Error bars in all panels represent SEM. The number following the compound abbreviation indicates the dose in mg/kg/day (unless otherwise indicated, ^∗^p < 0.05, ^∗∗^p < 0.01, and ^∗∗∗^p < 0.001, compared with no treatment (one-way ANOVA). (A) SEQ-9 dose-ranging in an acute TB mouse model. CFU counts after 4 weeks of treatment. Day 3 indicates lung CFU counts at treatment initiation (3 days after infection). (B) SEQ-9 dose-ranging in the mouse model of chronic TB: CFU counts after 4 weeks of treatment. Day 3 indicates lung CFU 3 days after infection. Day 31 indicates lung CFU counts at treatment initiation (28 days after infection). A PEG200/Tween80/citrate buffer (PEG-Tw) 100 mM (70/5/27) pH 4 formulation was used for SEQ-9, whereas MCT was used for rifampicin (RIF) and linezolid (LIN) (^∗^p < 0.05 and ^∗∗^p < 0.01, compared with no treatment. Kruskal-Walllis test). (C) SEQ-9 dose-ranging in short (highly) acute mouse model of TB. Day 1 indicates lung CFU counts at treatment initiation (1 day after infection). SEQ-9 (Lip/2) and control (day 11 Lip/2) groups are from an independent study using a lipidic formulation described in Figure S8 and Table S7 and were compared by Student’s t test: ###p < 0.001.

Based on pharmacokinetic (PK) studies that indicate the potential for efficacy with once-daily dosing (Table S7), the efficacy of SEQ-9 was evaluated in acute and chronic TB mouse models. SEQ-9 was orally administered in a methylcellulose-Tween-based formulation at daily doses (5 days per week) ranging from 37.5 to 300 mg/kg for 4 weeks in a murine model of acute TB. Mice were infected with 4.5 log_10_ CFU of actively replicating Mtb H37Rv strain 3 days before the beginning of treatment. All untreated control mice died within 4 weeks, whereas mice treated with SEQ-9 survived at all doses administered. Over the course of the treatment, the lung CFU counts decreased in a dose-dependent manner. Also, complete prevention of bacterial growth was observed in mice treated with 300 mg/kg of SEQ-9, thus defining the minimum effective dose (MED; Figure 5A).

To evaluate the efficacy of SEQ-9 in a murine chronic TB infection model, mice were infected with 2.3 log_10_ CFU of Mtb Erdman strain by aerosol administration, and treatment began 1 month after infection when the CFU in lung had stabilized at 6 log_10_. SEQ-9 was administered orally once daily in a PEG200/Tween 80/citrate buffer 100 mM (70/5/25) pH 4 formulation, at doses ranging from 75 to 300 mg/kg. SEQ-9 displayed dose-dependent bactericidal activity from 75 mg/kg with a significant 1.7 log CFU reduction at 300 mg/kg, compared with pre-treatment CFU. Importantly, the activity of SEQ-9 at 150 mg/kg was similar to that of linezolid at 100 mg/kg (Figure 5B).

Finally, SEQ-9 was evaluated in a mouse model of highly acute TB (Figure 5C), with treatment beginning 1 day after intranasal infection with 6.3 log_10_ CFU of Mtb H37Rv. SEQ-9 at 300 mg/kg reduced the mean lung CFU by 1.42 log_10_ after 2 weeks of treatment, almost achieving MED. In a second experiment for this model, a lipid-based formulation was used, and this improved bioavailability (Table S7). MED was reached at 100 mg/kg with a 2 log_10_ decrease in CFU.

Across these multiple efficacy studies, the monotherapy data clearly demonstrates dose dependency as well as net bacteriostatic effect against acute murine infection models and a net bactericidal effect against a chronic murine infection model. As suggested by the use of a lipid-based formulation in one model, dosing to reach MED can still be optimized. Although SEQ-9 doses and formulations differed across efficacy studies, taken together, the monotherapy data clearly demonstrate dose dependency and a net bacteriostatic effect against acute murine infection models and a net bactericidal effect against a chronic murine infection model. This is in line with previously published results for rifampicin at doses of 10–15 mg/kg/day, which routinely demonstrate bacteriostatic activity against acute TB models and bactericidal activity against chronic TB models,^30^^,^^31^^,^^32^ as seen in our chronic TB model (Figure 5). Linezolid^30^ and other oxazolidinone translation inhibitors such as AZD5847^32^ exhibit this profile as well. This behavior may be generalizable to translation and transcription inhibitors, given that rifampicin inhibits RNA polymerase. Of interest, bactericidal contributions of rifampicin, oxazolidinones, and SEQ-9 are seen in combinations of TB drugs.^8^^,^^33^^,^^34^^,^^35^

**Figure 6:**
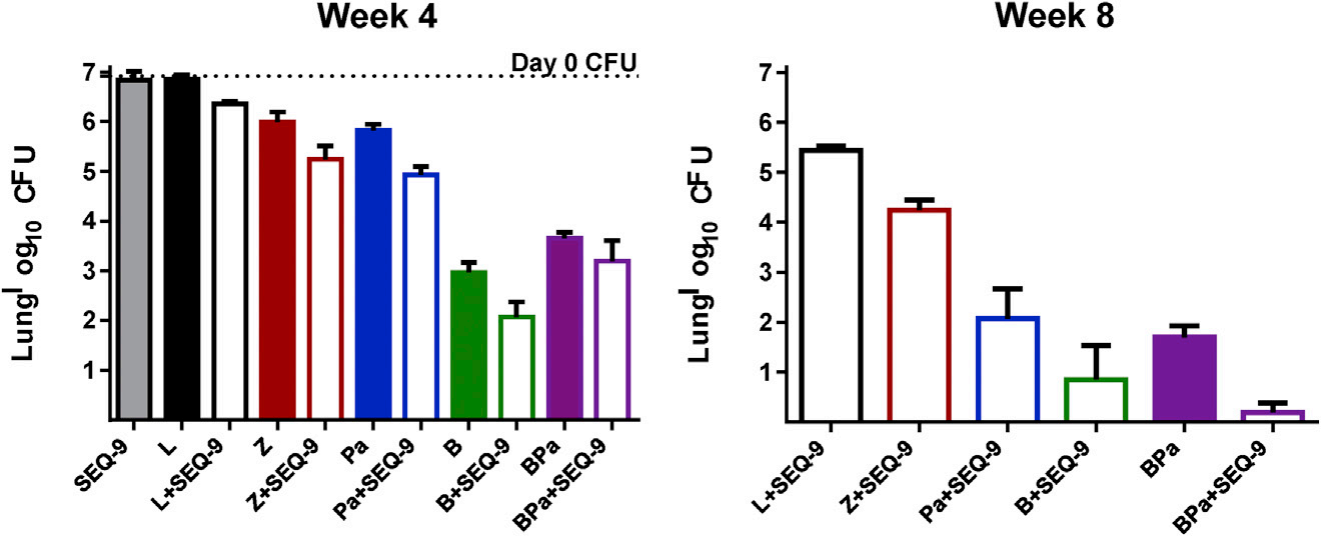
SEQ-9 administered in combination with anti-TB drugs during the intensive phase of treatment in a mouse model of TB Data represent the mean lung log_10_ CFU counts (4– 5 mice per group). Drug doses: SEQ-9 300 mg/kg/day, bedaquiline (B) 25 mg/kg/day, pretomanid (Pa) 100 mg/kg/day; linezolid (L) 100 mg/kg/day; pyrazinamide (Z) 150 mg/kg/day. Error bars in all panels represent standard deviations. Dotted bar indicates the mean CFU count in the lungs at the start of treatment (day 0).

The promising activity of SEQ-9 led us to evaluate its contribution to the efficacy against murine TB in drug combination therapies. To this end, mice were aerosol-infected with approximately 4 log_10_ CFU of Mtb H37Rv, and treatment started 2 weeks later. When administered for 4 weeks as a monotherapy, 300 mg/kg of SEQ-9 demonstrated bacteriostatic activity and prevented death, similarly to linezolid at 100 mg/kg. However, when combined with bedaquiline (B), pretomanid (Pa), linezolid (L), or pyrazinamide (Z), SEQ-9 contributed to bactericidal activity. Addition of SEQ-9 to B or Pa led to an additional reduction of approximately 1 log_10_ CFU, compared with B or Pa alone (p < 0.0001). When SEQ-9 was added to L or Z, lung CFU was reduced by approximately 0.5 log units more than L (p < 0.05) or Z (p < 0.001) alone. After 8 weeks of dosing, a notable acceleration of the bactericidal effect of Pa/SEQ-9 and B/Pa/SEQ-9 between weeks 4 and 8 was evident. The three-drug combination of B/Pa/SEQ-9 reduced the mean log CFU count to 0.2, compared with 1.69 for B/Pa in the absence of SEQ-9 (p < 0.01), demonstrating the substantial contribution of SEQ-9 to the bactericidal activity of B/Pa (Figure 6; Table S8).

## Discussion

Sequanamycin A is a naturally occurring macrolide that garnered significant interest in the 1960s as a potential TB treatment but was never fully developed. The current need for novel antibiotics has revived our interest in this compound as an Mtb-specific agent. Sequanamycins are unique among macrolides in that they demonstrate potent activity against Mtb, unlike the rest of the erythromycin-class macrolides. Like clarithromycin, they interact with the ribosomal peptide exit tunnel, but there are sufficient structural differences to explain the divergent potency of sequanamycins, compared with the rest of the erythromycin-class macrolides. Indeed, methylation by *erm37* is induced by sequanamycin treatment, consistent with other macrolides, but it does not affect the inhibition by sequanamycin, as they show equal inhibitory activity against the native and methylated Mtb ribosome. The cryo-EM structure of the Mtb ribosome with SEQ-9 clearly shows that the molecular basis for the sequanamycins’ ability to maintain potency against the methylated ribosome is due to a slight repositioning of the methylated N6 of A2058 that would normally have steric overlap with the dimethyl group of the desosamine found in all macrolide antibiotics. Sequanamycins lack the bulky dimethyl group, and this coupled with the slightly different position of the N6 of A2058 allows the sequanamycin macrolide to bind to the pocket without a significant loss of affinity.

Using the co-crystal structures of the *T. thermophilus* ribosome with SEQ-569 and SEQ-977, we rationalized modifications that would improve not only potency but also ADME properties. These optimizations led to SEQ-9, which demonstrated potent *in vitro* activity against laboratory strains of replicating and non-replicating Mtb, Mtb residing inside macrophages and to multiple clinical isolates.

SEQ-9 was also effective against Mtb resistant to individual first- or second-line drugs, an important feature in a new anti-TB drug. The pharmacological activity of SEQ-9, together with its PKs in mice, shows it distributes well into lung tissue and could be well suited to once-daily oral dosing. In acute and chronic infection mouse models, the compound demonstrated *in vivo* efficacy as a single agent and in combination with linezolid, bedaquiline, pretomanid, or pyrazinamide. It is notable that SEQ-9 showed additive activity with all TB drugs tested in the mouse model of TB, suggesting that this class of macrolide has the potential to contribute to a broad range of TB drugs and regimens. A notable acceleration of the bactericidal effect between weeks 4 and 8 was demonstrated when combining SEQ-9 with bedaquiline and pretomanid. After 8 weeks of treatment, the three-drug combination of bedaquiline/pretomanid/SEQ-9 reduced the mean log CFU count to 0.2, compared with 1.69 for bedaquiline/pretomanid in the absence of SEQ-9 (p < 0.01), demonstrating the ability of SEQ-9 to add substantial bactericidal activity to bedaquiline/pretomanid in the last 4 weeks of treatment. Previous studies in the same mouse model demonstrate a similar contribution to bedaquiline/pretomanid by linezolid,^8^ resulting in the three-drug BPaL regimen that produced excellent treatment outcomes as a 6-month oral regimen in the treatment of XDR-TB in the Nix-TB trial.^4^ This suggests that the sequanamycin series should be further explored as a potential alternative to linezolid, which may enrich the therapeutic arsenal targeting the Mtb ribosome.

### Limitations of the study

There are several limitations inherent to the techniques we use in the research presented. Both X-ray crystal and cryo-EM structures are static produced by averaging three-dimensional densities of the ribosome: sequanamycin complex. Most of the dynamic information related to the binding events is not directly available using these methods and can only be inferred. Also, the resolution of the structures is such that we can only approximate the distance of non-covalent interactions, e.g., hydrogen bonds.

A second limitation of the study is in the predictions of the pharmacology, toxicity, and efficacy of the sequanamycins based on cell data and efficacy experiments using mouse models of disease. Although there is relatively good agreement between mouse and human efficacy for most TB drugs, it is impossible to know if this will hold true for sequanamycins.

## STAR★Methods

### Key resources table

**Table:** 

REAGENT or RESOURCE	SOURCE	IDENTIFIER
**Bacterial and virus strains**		

*Allokutzneria albata*	DSMZ	DSMZ 24416
Mycobacterium tuberculosis : H37Rv LuxAB	Institute for Tuberculosis Research, University of Illinois at Chicago	N/A
Mycobacterium tuberculosis H37Rv	ATCC	Cat# ATCC27294
Mycobacterium tuberculosis H37Rv-GFP	IPBS Toulouse	N/A
Mtb Erdman bacteria	ATCC	Cat# ATCC35801
KMA3899 Lineage 1	Institute for Tuberculosis Research, University of Illinois at Chicago	N/A
KMA4244 Lineage 2	Institute for Tuberculosis Research, University of Illinois at Chicago	N/A
KMA4439 Lineage 2	Institute for Tuberculosis Research, University of Illinois at Chicago	N/A
KMA1354 Lineage 3	Institute for Tuberculosis Research, University of Illinois at Chicago	N/A
KMA5282 Lineage 4	Institute for Tuberculosis Research, University of Illinois at Chicago	N/A
KMA5319 Lineage 4	Institute for Tuberculosis Research, University of Illinois at Chicago	N/A
M. bovis BCG Pasteur	Institut Pasteur	IP1173P2
M. bovis Moreau [TMC1013 (BCG Brazilian)]	ATCC	ATCC 35736
E. coli Top10 cells	Invitrogen	Cat#C404050
A2296G mutants	This paper	N/A
Del 1848G mutant	This paper	N/A
Del 872G mutant	This paper	N/A
Del 874T	This paper	N/A

**Biological samples**		

Human liver microsomes	BD Gentest	Cat#452117
S30 fraction from *Mtb*	This paper	N/A
S100 fraction from *Mtb*	This paper	N/A
nanoluciferase mRNA	Cui et al.^36^	N/A

**Chemicals, peptides, and recombinant proteins**		

Sequanamycin A, (3S,4S,5R,7S,9S,10S,11R,12S,13R)-12-[(4,5-dihydroxy-4,6-dimethyltetrahydro-2H-pyran2-yl)oxy]-7-hydroxy-2-{1-[(5-hydroxy-3,4-dimethoxy-6-methyltetrahydro-2H-pyran-2- yl)oxy]propan-2-yl}-10-[(3-hydroxy-6-methyl-4-oxotetrahydro-2H-pyran-2-yl)oxy]- 3,5,7,9,11,13-hexamethyl-6,14-dioxooxacyclotetradecan-4-yl) 3-methylbutanoate.	Arnoux et al.^37^	CAS 1574520-92-5
Middlebrook 7H9 broth medium	Difco BD	Cat# 271310
Middlebrook 7H11 agar	Difco BD	Cat# 28381
Middlebrook 7H10 agar	Difco BD	Cat# 11799042
Glycerol	Prolabo	Cat# 24387
Palmitate	Sigma-Aldrich	Cat# P0500
Catalase	Sigma-Aldrich	Cat# C1345
Bovin Serum Albumin	Sigma-Aldrich	Cat# A3059
Oleic Albumin Dextrose Catalase (OADC)	BD	Cat# 21224
Casitone	Bacto BD	Cat# 22593
Tween 80	Sigma-Aldrich	Cat# P1754
DMSO (DiMethylSulphOxide)	Sigma-Aldrich	Cat# D2650
Phosphate- Buffered Saline (PBS)	Gibco	Cat# 041.94855A
Isoniazid	Sigma-Aldrich / ATCC	Cat# I3377/ ATCC-35838
Rifampicine	Sigma-Aldrich / ATCC	Cat# 83907/ ATCC-35822
Clarithromycin	Sigma-Aldrich	Cat# C9742
kanamycin	Sigma-Aldrich / ATCC	Cat# K1377/ ATCC-35827
Cycloserin	ATCC	ATCC-35826
Amikacin	Sigma-Aldrich	Cat# A1774
PA-824/ Pretonamid	Sanofi	N/A
Bedaquiline	Sanofi	N/A
Moxifloxacin	Alfa-Aesar	Cat# J66626
Linezolid	Sigma-Aldrich	Cat #PZ0014
Midazolam	BD Gentest	Cat#451028
Testosterone	Sigma-Aldrich	Cat# 86500
Ketoconazole	BD Gentest	Cat#451023
Azithromycin	Sigma-Aldrich	Cat# 75199
Erythromycin	Sigma-Aldrich	Cat# E5389
Clarithromycin	Sigma-Aldrich	Cat# C9742
Tylosin	Sigma-Aldrich	Cat# T6271
Clindamycin	Sigma-Aldrich (hydrochloride)	Cat#C5269
Nano-luciferase substrate Furimazine	Promega	Cat#N113
DMEM high glucose Glutamax 1X	Gibco	Cat#61965
SOC medium (Sigma)	Invitrogen	Cat#15544034
Fetal calf serum (Serum : Hyclone FetalClone II)	GE Healthcare Life Sciences	Cat# SH 30066-03
Crémophor RH40	Sigma	Cat #07076
Capryol 90	Gattefossé	N/A
Miglyol 812N	IOI Oleochemica	N/A

**Critical commercial assays**		

CellTiter-Blue	Promega	Cat# G8080
Cell Mask Blue	Invitrogen	Cat# H32720
Maxwell RSC Cultured Cells DNA Kit	Promega	Cat#AS1620
Maxwell RSC simply RNA Cells kit	Promega	Cat#AS1390
SuperScript™ IV VILO	Applied Biosystems	Cat#11756500

**Deposited data**		

70S Thermus thermophilus ribosome with bound antibiotic lead SEQ-569	This Paper	RCSB PDB: 7AZS (https://www.rcsb.org/structure/7AZS)
70S thermus thermophilus ribosome with bound antibiotic lead SEQ-977	This Paper	RCSB PDB: 7AZO (https://www.rcsb.org/structure/7AZO)
Methylated *Mtb* Ribosome 70S with Seq-9 Cryo-EM map	This Paper	EMDB: EMDB-22865 (https://www.ebi.ac.uk/emdb/EMD-22865)
Methylated *Mtb* Ribosome 70S with Seq-9 Cryo-EM atomic model	This Paper	PDB: 7KGB (https://www.rcsb.org/structure/7KGB)
Unmethylated *Mtb* Ribosome 50S with Seq-9 Cryo-EM map	This Paper	EMDB: EMDB-25100 (https://www.ebi.ac.uk/emdb/EMD-25100)
Unmethylated *Mtb* Ribosome 50S with Seq-9 Cryo-EM atomic model	This Paper	PDB: 7SFR (https://www.rcsb.org/structure/7SFR)
Structure of the Thermus thermophilus ribosome complexed with erythromycin	Bulkley et al.^13^	RCSB PDB: 4V7X (https://www.rcsb.org/structure/4V7X)
Crystal structure of the E. coli ribosome bound to erythromycin	Dunkle et al.^27^	RCSB PDB: 4V7U (https://www.rcsb.org/structure/4V7U)
Crystal structure of the A2058-unmethylated Thermus thermophilus 70S ribosome in complex with erythromycin and protein Y (YfiA) at 2.55A resolution	Svetlov et al.^26^	RCSB PDB: 6XHX (https://www.rcsb.org/structure/6XHX)
Cryo-EM structure of the 70S ribosome from Mycobacterium tuberculosis bound with Capreomycin	Yang et al.^38^	RCSB PDB: 5V93 (https://www.rcsb.org/structure/5V93)

**Experimental models: Cell lines**		

RAW264.7 murine macrophage-like cells	ATCC	ATCC #TIB-71

**Experimental models: Organisms/strains**		

Mouse: male Swiss mice	Janvier Laboratories	N/A
Mouse: female BALB/c	Charles River	N/A
Mouse: female BALB/c	Janvier Laboratories	N/A

**Oligonucleotides**		

erm37 was PCR amplified using oligonucleotides CTCGATCATATGGTGTCCGCCCTCGGACGGTC CATACTAAGCTTTTACC GCCCCTGCCAGTCAC ATCGTCGCGGCCGCGATATCGCCTCATTGGC	This paper	N/A
Taqman probes for detection of erm37 RNA F: AGCGATTCCCTGGCATTACC R: GCGGGTTCGCCACAAC FAM, ACGCCGCCTCGATCC)	This paper	N/A
Taqman probes for detection of 23S RNA F: GCGCCCGTGACGAATC R: ACGCAGCCCCAGAACTC FAM: TAACCACCCAAAACCG	This paper	N/A
The Rv0560c promoter was PCR amplified using oligonucleotidesATCGTTGACGTCGCGG CCGCATCGTGGGTTGCGGATGAGCATCGCT GAATTCGTGTTCATATATATCAACGGC	This paper	N/A

**Software and algorithms**		

PYMOL	The PyMOL Molecular Graphics System, Schrodinger	https://pymol.org/2/
Schrodinger Suite 2021-3	Schrodinger inc.	https://www.schrodinger.com/
Prism 8	GraphPad	https://www.graphpad.com/scientificsoftware/prism/
Xcalibur version 1.4 software	Thermo Fisher	https://thermo.flexnetoperations.com/control/thmo/
cryoSPARC	Punjani et al.^39^	https://cryosparc.com
Chimerax	Pettersen et al., 2021	https://www.rbvi.ucsf.edu/chimerax/
Chimera	Pettersen et al.^40^	https://www.cgl.ucsf.edu/chimera/
Coot	Emsley et al.^41^	https://www2.mrc-lmb.cam.ac.uk/personal/pemsley/coot/
Phenix	Afonine et al.^42^	http://www.phenix-online.org/
Relion	Scheres^43^	https://relion.readthedocs.io/en/release-3.1/
MotionCor2	Zheng et al.^44^	https://emcore.ucsf.edu/ucsf-software
Gctf	Zhang^45^	https://www2.mrc-lmb.cam.ac.uk/download/gctf/
Gautomatch	Kai Zhang	http://www.mrc-lmb.cam.ac.uk/kzhang/
MATLAB R2021a	MathWorks®	https://www.mathworks.com/products/new_products/release2021a.html

**Other**		

Plate reader	Perkin Elmer	Envision
fluorescent microscope HCS reader Cellomics ArrayScan VTI	Thermo Fisher	Cellomics ArrayScan VTI
RNA extraction: MaxWell RSC Instrument	Promega	Cat#AS4500
Grinder:Fast prep instrument	MP Biomedicals	FastPrep-24™ Classic Instrument
7500 Real-Time PCR system	Applied Biosystems	Cat#4351105
TSQ Quantum Ultra EMR MS detector	Thermo Fisher	N/A
Phenomenex Luna C5	Phenomenex	N/A
a Titan Krios electron microscope	Thermo Fisher	
Vitrobot Mark III	FEI company	
Gatan K2 Summit	Gatan	

### Resource availability

#### Lead contact

Further information and requests for resources and reagents should be directed to and will be fulfilled by the lead contact, James C. Sacchettini (sacchett@tamu.edu).

#### Materials availability

Bacterial cells A2296G mutants, Del 1848G mutant, Del 872G mutant, Del 874T and Plasmids generated in this study will be made available on request, but we may require a payment and/or a completed Materials Transfer Agreement if there is potential for commercial application.

All data to support the conclusions of this manuscript are included in the main text, supplemental information, and the Protein Data Bank (PDB: 7KGB), (PDB: 7AZO), (PDB: 7AZS), (PDB: 7SFR); and the Electron Microscopy Data Bank (EMDB: EMD-22865), (EMDB: EMD-25100)

### Experimental model and subject details

#### Cell culture

• E. coli Top10 cells (Invitrogen) were used for cloning experiments.
• RAW264.7 murine macrophage-like cells (ATCC #TIB-71) were grown at 37 °C with 5% CO2 in Dulbecco’s modified Eagle medium supplemented with 10% fetal calf serum, 4 mM L-glutamine and 1 mM sodium pyruvate.
• Green fluorescent protein (GFP)-expressing Mtb H37Rv were cultured at 37 °C and 5% CO2 in Middlebrook 7H9 medium supplemented with 10% (v/v) oleic acid-albumin-dextrose catalase (OADC), 0.4% (v/v) glycerol and 0.05% (v/v) Tween 80.
• Mtb H37Rv strain used in the MABA assay were cultured in Middlebrook 7H12 broth (Middlebrook 7H9 broth containing 0.1% w/v casitone, 5.6 μg/mL palmitic acid, 5 mg/mL bovine serum albumin, 4 μg/mL catalase), incubated at 37 °C and 5 % CO2 in plastic bags.
• Mtb strain H37Rv containing the inducible luciferase reporter plasmid pFCA-luxAB (LuxAB) was cultured in Middlebrook 7H12 medium supplemented with 25% of mouse or human serum incubated at 37 °C and 5 % CO2 in plastic bags.
• M. bovis BCG Pasteur (IP1173P2) and Moreau (TMC1013 (BCG Brazilian), ATCC35736) strains were cultivated in Middlebrook 7H9 broth supplemented with 10% (v/v) OADC, 0.4% (v/v) glycerol and 0.05% (v/v) Tween 80 at 37°C and 5% CO2.

#### Organisms

• Male Swiss mice (Janvier Laboratories, Saint Isle, France). Mice were fed ad libitum.
• Female BALB/c mice aged 5 to 7 weeks were purchased from Janvier Laboratories, France. Mice were housed at 5 animals per cage with sterile bedding in HEPA–filtered racks (Tecniplast, France) in certified animal biosafety level 3 (ABSL-3) laboratories. Temperatures were maintained at 65-75°F (∼18-23 °C) with 40-60% humidity. Specific pathogen-free status was verified by testing sentinel mice housed within the same rack. Mice had ad libitum access to water and food pellets.
• For combination studies, female BALB/c mice 6-8 weeks old, purchased from Charles River (Wilmington, MA, USA) were used.

### Method details

#### General synthetic scheme to Sequanamycin derivatives

**Figure:**
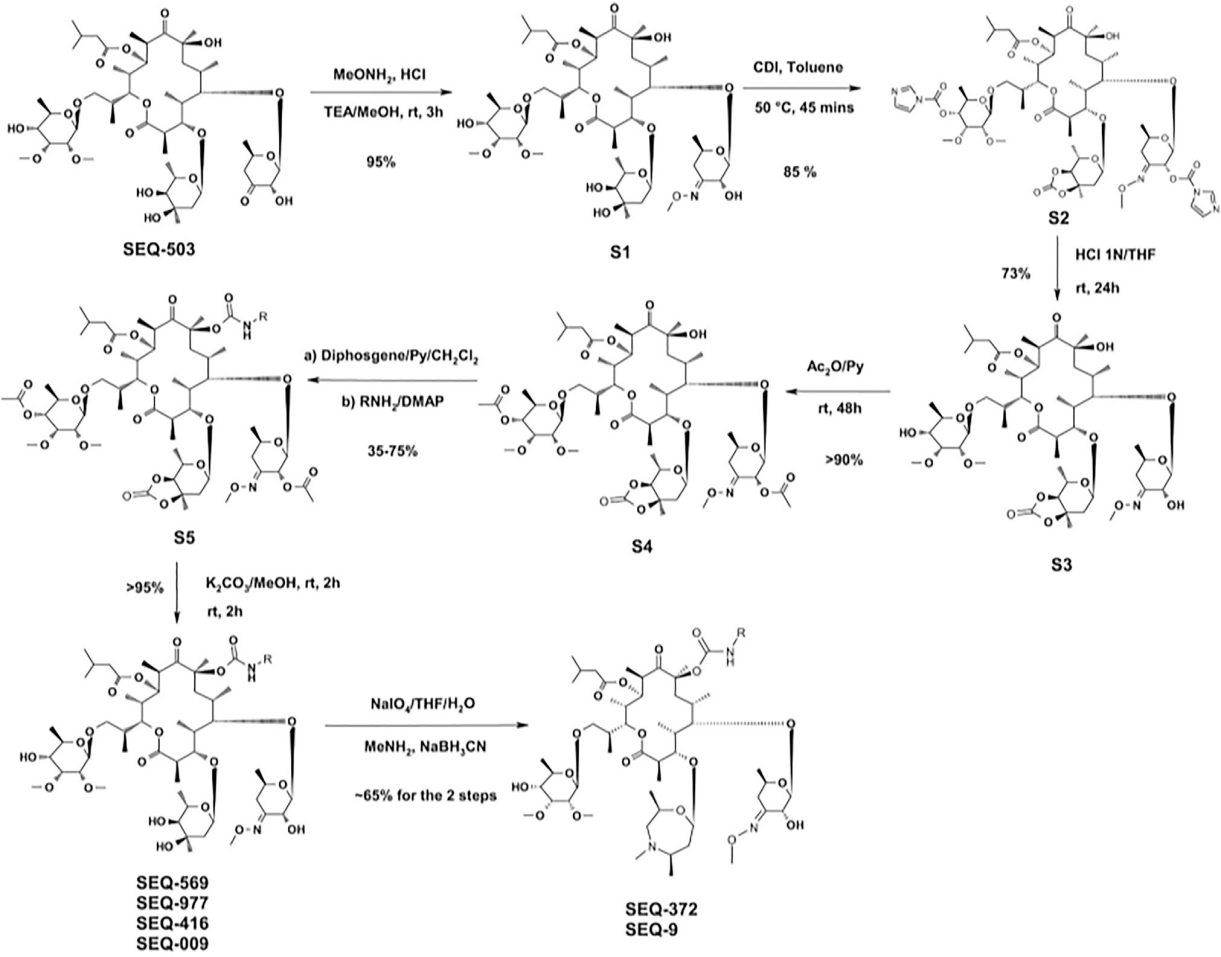

#### Materials and methods for the synthesis of Sequanamycin derivatives

The progress of the synthetic reactions was monitored by TLC using plates coated with Merck 60 F254 silica gel. After elution, the plates were observed under ultraviolet light at 254 nm and then revealed by spraying with a 5M sulfuric acid/water solution followed by heating. The microwave reactions were performed using a Biotage Initiator 8 EXP microwave machine. The products were purified, when necessary, on a Biotage SP-1 chromatograph or a Spot 2 chromatograph from Merck. The columns used are Merck 15-40 μm silica columns (2.5 g to 400 g).

In the Preparations and in the Examples, the following abbreviations are used:

EtOAc: ethyl acetate
TLC: thin-layer chromatography
CHCl3: chloroform
DCM: dichloromethane
DMF: N,N-dimethylformamide
TEA: triethylamine
NaIO4: sodium metaperiodate, sodium periodate
K2CO3: potassium carbonate
MeOH: methanol
MgSO4: magnesium sulfate
NaBH3CN: sodium cyanoborohydride
NaCl: sodium chloride
NaHCO3: sodium bicarbonate
Na2SO4: sodium sulfate
NH4Cl: ammonium chloride
NH4Ac: ammonium acetate
THF: tetrahydrofuran
rt: room temperature

##### Mass spectrometry (MS)

*Method a*. The spectra were acquired on a Waters UPLC-SQD

Ionization: electrospray in positive and/or negative mode (ES+/-);

Chromatographic conditions:

Column: Acquity BEH C18 - 1.7 μm - 2.1 x 50 mm,

Solvents: A: H2O (0.1% formic acid) B: CH3CN (0.1% formic acid),

Column temperature: 50°C,

Flow rate: 1 ml/min,

Gradient (2 min): from 5% to 50% B over 0.8 min; 1.2 min: 100% B; 1.85 min: 100% B; 1.95: 5% B.

*Method b*. The spectra were acquired on a Waters UPLC-SQD

Ionization: electrospray in positive and/or negative mode (ES+/-);

Chromatographic conditions:

Column: Acquity BEH C18 - 1.7 μm - 2.1 x 50 mm,

Solvents: A: H2O (0.1% formic acid) B: CH3CN (0.1% formic acid),

Column temperature: 50°C,

Flow rate: 0.8 ml/min,

Gradient (2.5 min): from 5% to 100% B over 1.8 min; 2.40 min: 100% B; 2.45 min: 100% B; from 100% to 5% B over 0.05 min.

*Method c*. The spectra were acquired on a Waters ZQ

Ionization: electrospray in positive and/or negative mode (ES+/-);

Chromatographic conditions:

Column: XBridge C18- 2.5 μm - 3 x 50 mm,

Solvents: A: H2O (0.1% formic acid) B: CH3CN (0.1% formic acid),

Column temperature: 70°C,

Flow rate: 0.9 ml/min,

Gradient (7 min): from 5% to 100% B over 5.3 min; 5.5 min: 100% B; 6.3 min: 5%

*Method d.* The spectra were acquired on a Waters XEVO ACQUITY UPLC

Ionization: electrospray in positive and/or negative mode (ES+/-);

Chromatographic conditions:

Column: HSS T3 1.8μm 2.1x100mm

Solvents: A: H2O (0.1% formic acid) B: CH3CN (0.1% formic acid),

Column temperature: 45°C,

Flow rate: 0.5 ml/min,

Gradient (11 min): from 5% to 97% B over 11 min

#### ^1^H Nuclear magnetic resonance (NMR)

The ^1^H NMR spectra were recorded on a Brüker Avance spectrometer (300 MHz, 400 MHz, 500 MHz or 600 MHz) in deuterated DMSO. The chemical shifts are expressed in units δ (ppm) using tetramethylsilane (TMS) as internal reference. For the interpretation of the spectra, the following abbreviations were used: s = singlet, d = doublet, t = triplet, q = quartet, quint = quintet, sext = sextet, dd = doubled doublet, ddd = doublet of doubled doublets, m = multiplet, ax. = axial, equat. = equatorial.

Synthesis of (2R,3S,4R,5R,7S,9S,10S,11R,12S,13R)-12-{[(2R,4R,5S,6S)-4,5-dihydroxy-4,6-dimethyltetrahydro-2H-pyran-2-yl]oxy}-7-hydroxy-2-(1-{[(2R,3R,4R,5R,6R)-5-hydroxy-3,4-dimethoxy-6-methyltetrahydro-2H-pyran-2-yl]oxy}propan-2-yl)-10-{[(2S,3R,6R)-3-hydroxy-4-(methoxyimino)-6-methyltetrahydro-2H-pyran-2-yl]oxy}-3,5,7,9,11,13-hexamethyl-6,14-dioxooxacyclotetradecan-4-yl 3-methylbutanoate (**S1**)

12 g of sequanamycin (A) were dissolved in 175 ml of MeOH with stirring, 5.3 ml of TEA, and 3 g of methylhydroxylamine hydrochloride were then added, in this order. The stirring was continued at rt for 20 hours and MeOH was then evaporated under vacuum. The crude reaction product was taken up in 150 ml of DCM and washed with 100 ml of water and then with 100 ml of saturated aqueous NaCl solution. The aqueous phases were extracted with 150 ml of DCM. The organic phases were combined, dried over MgSO4, filtered and concentrated under vacuum. 12.7 g of the product obtained were suspended in 70 ml of a petroleum ether (40-60°C)/isopropanol mixture (2/1). The mixture was heated to 70°C, the insoluble matter was filtered off while hot and the filtrate was then left to precipitate out at rt over 20 hours. The precipitate was filtered off by suction and rinsed with 20 ml of a petroleum ether (40-60°C)/isopropanol mixture (2/1, and dried under vacuum at 35°C to give 10.62 g of expected product.

MS: method c

Retention time Tr (min) = 4.87; [M+Na]^+^: m/z 1014; [M-H+HCO_2_H]^-^: m/z 1036.

^1^H NMR spectrum (500 MHz, in ppm, DMSO-d_6_): 0.81 (d, J=6.8 Hz, 3 H); 0.93 to 1.01 (m, 15 H); 1.07 (d, J=7.0 Hz, 3 H); 1.09 to 1.13 (m, 9 H); 1.17 (d, J=6.0 Hz, 3 H); 1.18 (d, J=6.0 Hz, 3 H); 1.24 (s, 3H); 1.44 (dd, J=10.8 and 14.4 Hz, 1 H); 1.68 to 1.76 (m, 2 H); 1.81 (d, J=14.4 Hz, 1 H); 1.88 (dd, J=11.5 and 15.9 Hz, 1 H); 1.96 to 2.06 (m, 3 H); 2.07 to 2.20 (m, 4 H); 2.73 (quint, J=7.0 Hz, 1 H); 2.81 (t, J=9.0 Hz, 1H); 2.89 to 2.97 (m, 2 H); 3.03 (ddd, J=2.5 and 7.3 and 9.5 Hz, 1 H); 3.18 (q, J=6.8 Hz, 1 H); 3.34 to 3.36 (m, 2 H); 3.37 (s, 3 H); 3.45 (s, 3 H); 3.52 (dq, J=6.2 and 9.4 Hz, 1 H); 3.60 (s, 1 H); 3.62 to 3.65 (m, 1 H); 3.66 (t, J=2.5 Hz, 1 H); 3.71 to 3.77 (m, 1 H); 3.78 (m, 1 H); 3.80 (s, 3 H); 3.81 to 3.84 (m, 1 H); 3.87 (m, 1 H); 4.39 to 4.46 (m, 3 H); 4.50 (s, 1 H); 4.72 (d, J = 8.3 Hz, 1 H); 4.78 (d, J=8.3 Hz, 1 H); 4.84 (d, J=7.3 Hz, 1 H); 4.87 (d, J=3.8 Hz, 1 H); 5.19 (d, J=4.4 Hz, 1 H).

Synthesis of [(2R,3R,4R,5R,6R)-6-[(2S)-2-[(2R,3S,4R,5R,7S,9S,10S,11R,12S,13R)-12-[[(3aS,4S,6R,7aR)-4,7a-dimethyl-2-oxo-3a,4,6,7-tetrahydro-[1,3]dioxolo[4,5-c]pyran-6-yl]oxy]-7-hydroxy-10-[(2S,3R,4E,6R)-3-(imidazole-1-carbonyloxy)-4-methoxyimino-6-methyl-tetrahydropyran-2-yl]oxy-3,5,7,9,11,13-hexamethyl-4-(3-methylbutanoyloxy)-6,14-dioxo-oxacyclotetradec-2-yl]propoxy]-4,5-dimethoxy-2-methyl-tetrahydropyran-3-yl] imidazole-1-carboxylate (S2):

11.1 g of the compound prepared in Preparation 1 were placed in 220 ml of toluene. 9.07 g of 1,1′-carbonyldiimidazole were added and the reaction medium was then heated at 60°C for 45 minutes. It was allowed to cool to rt and the precipitate was filtered off and washed with toluene. The toluene phase was washed with 100 ml of water and then dried over MgSO_4_. After filtration, the solvent was evaporated off to dryness and 14.26 g of the expected product were recovered.

MS: method a

Retention time Tr (min) = 1.22; [M+H]^+^: m/z 1206; [M-H+HCOOH]^-^: m/z 1250.

^1^H NMR spectrum (500 MHz, in ppm, DMSO-d_6_): 0.80 (d, J=6.8 Hz, 3 H); 0.83 (d, J=7.3 Hz, 3 H); 0.90 (d, J=6.8 Hz, 3 H); 0.98 (dt, J=3.2 and 6.4 Hz, 9 H); 1.03 (d, J = 7.3 Hz, 3 H); 1.13 (d, J=6.8 Hz, 3 H); 1.16 (d, J=6.4 Hz, 3 H); 1.24 (t, J=2.9 Hz, 6 H); 1.29 (d, J=5.9 Hz, 3 H); 1.53 (m, 4 H); 1.78 (m, 1 H); 1.84 to 1.91 (m, 1 H); 1.98 (m, 1 H); 2.02 to 2.11 (m, 3 H); 2.16 (m, 3 H); 2.21 (m, 1 H); 2.34 (dd, J=5.9 and 14.2 Hz,1 H); 2.73 (dq, J=7.2 and 7.3 Hz, 1 H); 3.08 to 3.17 (m, 3 H); 3.38 (m, 1 H); 3.41 (s, 3 H); 3.44 (s, 3 H); 3.56 (d, J=5.9 Hz, 1 H); 3.66 to 3.74 (m, 5 H); 3.78 (ddd, J=2.9 and 6.0 and 11.6 Hz, 1 H); 3.96 (m, 1 H); 4.07 to 4.20 (m, 3 H); 4.39 (s, 1 H); 4.58 (m, 2 H); 4.63 (d, J=9.3 Hz, 1 H); 4.76 (d, J=9.8 Hz, 1 H); 4.91 (s, 2 H); 5.22 (d, J=6.8 Hz, 1 H); 7.12 (d, J=10.3 Hz, 2 H); 7.61 (d, J=1.5 Hz, 2 H); 8.29 (d, J=8.8 Hz, 2 H).

Synthesis of [(2R,3S,4R,5R,7S,9S,10S,11R,12S,13R)-12-[[(3aS,4S,6R,7aR)-4,7a-dimethyl-2-oxo-3a,4,6,7-tetrahydro-[1,3]dioxolo[4,5-c]pyran-6-yl]oxy]-7-hydroxy-2-[(1S)-2-[(2R,3R,4R,5R,6R)-5-hydroxy-3,4-dimethoxy-6-methyl-tetrahydropyran-2-yl]oxy-1-methyl-ethyl]-10-[(2S,3R,4E,6R)-3-hydroxy-4-methoxyimino-6-methyl-tetrahydropyran-2-yl]oxy-3,5,7,9,11,13-hexamethyl-6,14-dioxo-oxacyclotetradec-4-yl] 3-methylbutanoate (**S3**):

14.01 g of the compound obtained in Preparation S2 was placed in 140 ml of THF, 34.8 ml of 1N HCl solution was added, and the mixture was stirred for 24 hours at rt. The reaction medium was poured into 200 ml of DCM and washed with 100 ml of water and then with 100 ml of saturated aqueous sodium bicarbonate solution. The aqueous phases were extracted with 200 ml of DCM, and the organic phases were combined, dried over Na_2_SO_4_, filtered and then evaporated to dryness under vacuum. 11.48 g of the expected product were obtained.

MS: method a

Retention time Tr (min) = 1.19; [M+Na]^+^: m/z 1040; [M-H + HCOOH]^-^: m/z 1062.

^1^H NMR spectrum (400 MHz, in ppm, DMSO-d_6_): 0.80 (d, J = 6.8 Hz, 3 H); 0.95 (m, 15 H); 1.03 to 1.17 (m, 12 H); 1.23 (s, 3 H); 1.30 (d, J = 5.9 Hz, 3 H); 1.45 (m, 1 H); 1.52 (s, 3 H); 1.78 to 1.89 (m, 2 H); 1.90 to 2.24 (m, 8 H); 2.37 (dd, J = 5.1 and 13.9 Hz, 1 H); 2.79 (m, 2 H); 2.91 (m, 1 H); 3.03 (ddd, J = 2.7 and 6.8 and 9.5 Hz, 1 H); 3.13 (q, J = 6.7 Hz, 1 H); 3.32 (masked m, 1 H); 3.37 (s, 3 H); 3.45 (s, 3 H); 3.52 (m, 2 H); 3.63 to 3.72 (m, 3 H); 3.80 (s, 3 H); 3.92 (t, J = 3.9 Hz, 1 H); 4.00 (ddd, J = 3.2 and 6.1 and 11.7 Hz, 1 H); 4.08 (m, 1 H); 4.15 (m, 1 H); 4.34 (s, 1 H); 4.45 (d, J = 8.1 Hz, 1 H); 4.63 (d, J = 9.8 Hz, 1 H); 4.68 (d, J=4.2 Hz, 1 H); 4.76 (d, J=9.5 Hz, 1 H); 4.83 (d, J = 7.1 Hz, 1 H); 4.98 (dd, J = 5.1 and 9.0 Hz, 1 H); 5.43 (d, J = 3.9 Hz, 1 H).

Synthesis of [(2R,3S,4R,5R,7S,9S,10S,11R,12S,13R)-12-[[(3aS,4S,6R,7aR)-4,7a-dimethyl-2-oxo-3a,4,6,7-tetrahydro-[1,3]dioxolo[4,5-c]pyran-6-yl]oxy]-2-[(1S)-2-[(2R,3R,4R,5R,6R)-5-acetoxy-3,4-dimethoxy-6-methyl-tetrahydropyran-2-yl]oxy-1-methyl-ethyl]-10-[(2S,3R,4E,6R)-3-acetoxy-4-methoxyimino-6-methyl-tetrahydropyran-2-yl]oxy-7-hydroxy-3,5,7,9,11,13-hexamethyl-6,14-dioxo-oxacyclotetradec-4-yl] 3-methylbutanoate (S4):

11.4 g of the compound obtained in Preparation S3 were placed in 114 ml of pyridine, and 7.4 ml of acetic anhydride were then added. The reaction medium was stirred at rt for 24 hours. The pyridine was evaporated off under vacuum and 200 ml of DCM were added to the crude reaction mixture. The resulting mixture was washed with 150 ml of 1N HCl solution and then with 150 ml of water. The aqueous phases were extracted with twice 200 ml of DCM, and the organic phases were combined, dried over Na_2_SO_4_, filtered and then evaporated to dryness under vacuum. 12.2 g of compound were obtained, which product were purified by chromatography on a Merck column (400 g of 15-40 μm silica), eluting with a 45/55 EtOAc/heptane mixture. 8.6 g of the expected product were recovered.

MS: method a

Retention time Tr (min) = 1.29; [M+H]^+^: m/z 1102; [M-H+HCOOH]^-^: m/z 1146.

^1^H NMR spectrum (400 MHz, in ppm, DMSO-d_6_): 0.81 (d, J=7.3 Hz, 3 H); 0.95 (m, 15 H); 1.06 (m, 6 H); 1.11 (d, J=6.6 Hz, 3 H); 1.20 (d, J=6.1 Hz, 3 H); 1.24 (s, 3 H); 1.29 (d, J=3.9 Hz, 3 H); 1.41 to 1.54 (m, 4H); 1.77 to 1.91 (m, 2 H); 1.96 to 2.09 (m, 10 H); 2.11 to 2.23 (m, 4 H); 2.36 (m, 1 H); 2.79 (m, 1 H); 3.02 (m, 2 H); 3.13 (m, 1 H); 3.35 (m, 1 H); 3.38 (s, 3 H); 3.41 (s, 3 H); 3.52 (d, J=7.1 Hz, 1 H); 3.64 to 3.73 (m, 2 H); 3.74 to 3.83 (m, 5 H); 3.86 (broad s, 1 H); 4.06 to 4.14 (m, 2 H); 4.33 to 4.40 (m, 2 H); 4.52 (d, J=8.1 Hz, 1 H); 4.65 (d, J=9.8 Hz, 1 H); 4.76 (m, 2 H); 4.97 (dd, J=5.3 and 8.9 Hz, 1 H); 5.02 (d, J=6.1 Hz, 1 H).

Synthesis of (2R,3S,4R,5R,7S,9S,10S,11R,12S,13R)-12-{[(2R,4R,5S,6S)-4,5-dihydroxy-4,6-dimethyltetrahydro-2H-pyran-2-yl]oxy}-2-(1-{[(2R,3R,4R,5R,6R)-5-hydroxy-3,4-dimethoxy-6-methyltetrahydro-2H-pyran-2-yl]oxy}propan-2-yl)-10-{[(2S,3R,6R)-3-hydroxy-4-(methoxyimino)-6-methyltetrahydro-2H-pyran-2-yl]oxy}-3,5,7,9,11,13-hexamethyl-7-({[2-(2-methyl-5-nitro-1H-imidazol-1-yl)ethyl]carbamoyl}oxy)-6,14-dioxooxacyclotetradecan-4-yl 3-methylbutanoate (SEQ-569)

Step 1) 4 g of the compound obtained in Preparation S4 were placed, under argon, in 190 ml of DCM, and 4.12 ml of pyridine were added. The solution was cooled to -20°C, followed by addition, in a single portion, of 0.8 ml diphosgene, and stirring was continued at -20°C for 3 hours. 0.443 g of 4-dimethylaminopyridine were added, while still at -20°C, the mixture was then allowed to warm to rt and stirred for 20 hours. The DCM was evaporated off under vacuum and the crude reaction product was taken up in 150 ml of EtOAc and stirred for 1 hour at rt. The precipitate formed was filtered off and rinsed with 80 ml of EtOAc. The filtrate was evaporated to dryness under vacuum, 4.7 g of the expected compound was used as for the following stage.

Step 2) 0.5 g (0.388 mmol) of the compound, prepared in step 1, dissolved in 14 ml of DMF were placed in a 30 ml round-bottomed flask, to which were added 0.378 g (1.52 mmol) of 2-(2-Methyl-5-nitroimidazol-1-yl)ethylamine dihydrochloride and 426μL of TEA, The mixture was stirred for 24 hours at rt, and 20 g of ice and 20 ml of water were then added. The precipitate formed was drained by suction and washed with a minimum amount of water. The crude reaction product (0.74 g) was purified by chromatography on a Merck column (50 g of 15-40 μm silica), eluting with a 75/25 EtOAc/heptane mixture. 0.3 g of expected product was recovered.

MS: Method a

Retention time Tr (min) = 1.09; [M+H]^+^: m/z 1188; [M-H]^-^: m/z 1186; [M-H+HCO2H]^-^: m/z 1232

^1^H NMR Spectrum (500 MHz, δ in ppm, DMSO-d_6_): 0.80 (d, J = 6.9 Hz, 3 H); 0.90 (d, J = 6.9 Hz, 3 H); 0.96 (m, 6 H); 0.99 (d, J = 6.9 Hz, 3 H); 1.02 to 1.07 (m, 9 H); 1.09 (s, 3 H); 1.12 (d, J = 6.0 Hz, 3 H); 1.17 (m, 6 H); 1.58 to 1.78 (m, 4 H); 1.65 (s, 3 H); 1.80 to 1.92 (m, 2 H); 1.94 to 2.07 (m, 3 H); 2.12 (m, 1 H); 2.17 (d, J = 6.9 Hz, 2 H); 2.41 (s, 3 H); 2.62 (m, 1 H); 2.80 (t, J = 9.1 Hz, 1 H); 2.92 (dd, J = 2.5 and 8.0 Hz, 1H); 2.97 (m, 2 H); 3.04 (m, 1 H); 3.20 to 3.35 (partially masked m, 3 H); 3.38 (s, 3 H); 3.40 (m, 1 H); 3.46 (s, 3 H); 3.53 (m, 1 H); 3.60 (s, 1 H); 3.63 (dd, J = 4.4 and 9.9 Hz, 1 H); 3.67 (t, J = 2.5 Hz, 1 H); 3.70 (m, 1 H); 3.79 (m, 1 H); 3.80 (s, 3 H); 3.88 (t, J = 5.1 Hz, 1 H); 4.02 (broad d, J = 3.6 Hz, 1 H); 4.17 to 4.38 (m, 4 H); 4.44 (d, J = 8.0 Hz, 1 H); 4.48 (d, J = 9.6 Hz, 1 H); 4.54 (d, J = 9.3 Hz, 1 H); 4.83 (d, J = 7.1 Hz, 1H); 4.91 (broad d, J = 3.0 Hz, 1 H); 5.12 (d, J = 5.1 Hz, 1 H); 6.93 (t, J = 5.9 Hz, 1 H); 8.00 (s, 1 H)

Step 3) The compound isolated in step 2 (0.25 g; 0.192 mmol) was reacted in 5 ml of methanol in the presence of sodium carbonate (0.133 g; 0.963 mmol, The heterogeneous medium was stirred at rt for 3 hours and then filtered through a No. 4 sinter funnel. The filtrate was taken up in 100 ml of EtOAc and washed with saturated aqueous NaCI solution. The organic phase was dried over MgSO4, filtered and then evaporated to dryness. 0.24 g of compound was obtained, which was purified by chromatography on a Merck column (15 g of 15-40 μm silica), eluting with a 95/5 CH_2_Cl_2_/isopropanol mixture. 0.169 g of expected product SEQ-569 was recovered.

MS: Method a

Retention time Tr (min) = 1.09; [M+H]^+^: m/z 1188; [M-H]^-^: m/z 1186; [M-H+HCO2H]^-^: m/z 1232 (base peak)

^1^H NMR Spectrum (500 MHz, δ in ppm, DMSO-d_6_): 0.80 (d, *J* = 6.9 Hz, 3 H); 0.90 (d, *J* = 6.9 Hz, 3 H); 0.96 (m, 6 H); 0.99 (d, *J* = 6.9 Hz, 3 H); 1.02 to 1.07 (m, 9 H); 1.09 (s, 3 H); 1.12 (d, *J* = 6.0 Hz, 3 H); 1.17 (m, 6 H); 1.58 to 1.78 (m, 4 H); 1.65 (s, 3 H); 1.80 to 1.92 (m, 2 H); 1.94 to 2.07 (m, 3 H); 2.12 (m, 1 H); 2.17 (d, *J* = 6.9 Hz, 2 H); 2.41 (s, 3 H); 2.62 (m, 1 H); 2.80 (t, *J* = 9.1 Hz, 1 H); 2.92 (dd, *J* = 2.5 and 8.0 Hz, 1 H); 2.97 (m, 2 H); 3.04 (m, 1 H); 3.20 to 3.35 (partially masked m, 3 H); 3.38 (s, 3 H); 3.40 (m, 1 H); 3.46 (s, 3 H); 3.53 (m, 1 H); 3.60 (s, 1 H); 3.63 (dd, *J* = 4.4 and 9.9 Hz, 1 H); 3.67 (t, *J* = 2.5 Hz, 1 H); 3.70 (m, 1 H); 3.79 (m, 1 H); 3.80 (s, 3 H); 3.88 (t, *J* = 5.1 Hz, 1 H); 4.02 (broad d, *J* = 3.6 Hz, 1 H); 4.17 to 4.38 (m, 4 H); 4.44 (d, *J* = 8.0 Hz, 1 H); 4.48 (d, *J* = 9.6 Hz, 1 H); 4.54 (d, *J* = 9.3 Hz, 1 H); 4.83 (d, *J* = 7.1 Hz, 1 H); 4.91 (broad d, *J* = 3.0 Hz, 1 H); 5.12 (d, *J* = 5.1 Hz, 1 H); 6.93 (t, *J* = 5.9 Hz, 1 H); 8.00 (s, 1 H).

Synthesis of [(2R,3S,4R,5R,7S,9S,10S,11R,12S,13R)-12-[(2R,4R,5S,6S)-4,5-dihydroxy-4,6-dimethyl-tetrahydropyran-2-yl]oxy-2-[(1S)-2-[(2R,3R,4R,5R,6R)-5-hydroxy-3,4-dimethoxy-6-methyl-tetrahydropyran-2-yl]oxy-1-methyl-ethyl]-10-[(2S,3R,4E,6R)-3-hydroxy-4-methoxyimino-6-methyl-tetrahydropyran-2-yl]oxy-3,5,7,9,11,13-hexamethyl-7-[2-[(2-nitrophenyl)sulfonylamino]ethylcarbamoyloxy]-6,14-dioxo-oxacyclotetradec-4-yl] 3-methylbutanoate (SEQ-977)

same procedure as SEQ-569, from **S4**, was used while replacing 2-(2-Methyl-5-nitroimidazol-1-yl)ethylamine dihydrochloride with 1-amino-2-(2-nitrobenzenesulfonamido)ethane

MS : method a

Retention time Tr (min) = 1.14; [M+Na]^+^ : m/z 1285; [M-H]^-^ : m/z 1261

^1^H NMR Spectrum (400 MHz, δ in ppm, DMSO-d_6_): 0.79 (d, J=6,8 Hz, 3 H); 0.89 to 0.96 (m, 9 H); 1.00 (d, J=6.8 Hz, 3 H); 1.02 to 1.13 (m, 15 H); 1.17 (m, 6 H); 1.62 to 2.07 (m, 12 H); 2.13 (m, 3 H); 2.59 to 2.66 (m,1 H); 2.81 (t, J=9.0 Hz, 1 H); 2.86 to 3.11 (m, 8 H); 3.2 (masked m, 1 H); 3.37 (s, 4 H); 3.46 (s, 3 H); 3.48 to 3.56 (m, 1 H); 3.58 to 3.72 (m, 4 H); 3.75 to 3.83 (m, 4 H); 3.87 (t, J=5.4 Hz, 1 H); 4.02 (broad s, 1 H); 4.33 to 4.39 (m, J=8.3 Hz, 2 H); 4.44 (d, J=8.3 Hz, 1 H); 4.49 (d, J=10.3 Hz, 1 H); 4.53 (d, J=9,3 Hz, 1 H); 4.85 (d, J=7.3 Hz, 1 H); 4.92 (broad s, 1 H); 5.12 (d, J=5.4 Hz, 1 H); 6.75 (t, J=5.1 Hz, 1 H); 7.82 to 7.90 (m, 2 H);7.94 to 8.02 (m, 2 H); 8.06 (d, J=2.4 Hz, 1 H)

Synthesis of [(2R,3S,4R,5R,7S,9S,10S,11R,12S,13R)-7-(benzylcarbamoyloxy)-12-[(2R,4R,5S,6S)-4,5-dihydroxy-4,6-dimethyl-tetrahydropyran-2-yl]oxy-2-[(1S)-2-[(2R,3R,4R,5R,6R)-5-hydroxy-3,4-dimethoxy-6-methyl-tetrahydropyran-2-yl]oxy-1-methyl-ethyl]-10-[(2S,3R,4E,6R)-3-hydroxy-4-methoxyimino-6-methyl-tetrahydropyran-2-yl]oxy-3,5,7,9,11,13-hexamethyl-6,14-dioxo-oxacyclotetradec-4-yl] 3-methylbutanoate (**SEQ-416**)same procedure as SEQ-569, from **S4**, was used while replacing 2-(2-Methyl-5-nitroimidazol-1-yl)ethylamine dihydrochloride with benzylamine.

MS: Method c

Retention time Tr (min) = 5.21; [M+H]^+^: m/z 1125; base peak: m/z 981 [M-H+HCO2H]^-^: m/z 1169

^1^H NMR Spectrum (500 MHz, δ in ppm, DMSO-d_6_): 0.80 (d, *J =* 6.8 Hz, 3 H); 0.92 (d, *J =* 6.8 Hz, 3 H); 0.96 to 1.02 (m, 9 H); 1.05 (m, 6 H); 1.08 to 1.15 (m, 9 H); 1.18 (m, 6 H); 1.67 to 2.18 (m, 10 H); 1.73 (s, 3 H); 2.18 (d, *J =* 6.8 Hz, 2 H); 2.59 to 2.67 (m, 1 H); 2.80 (t, *J =* 8.8 Hz, 1 H); 2.92 (dd, *J =* 2.7 and 8.1 Hz, 1 H); 2.94 to 3.06 (m, 3 H); 3.27 to 3.35 (partially masked m, 1 H); 3.38 (s, 3 H); 3.41 (d, *J =* 9.8 Hz, 1 H); 3.45 (s, 3 H); 3.49 to 3.56 (m, 1 H); 3.60 to 3.72 (m, 4 H); 3.80 (s, 3 H); 3.82 (m, 1 H); 3.87 (broad d, *J =* 5.4 Hz, 1 H); 4.01 to 4.17 (m, 3 H); 4.34 to 4.39 (m, 2 H); 4.45 (d, *J =* 7.8 Hz, 1 H); 4.50 to 4.57 (m, 2 H); 4.85 (d, *J =* 7.3 Hz, 1 H); 4.93 (d, *J =* 2.4 Hz, 1 H); 5.13 (broad s, 1 H); 7.18 to 7.36 (m, 6 H)

Synthesis of [(2R,3S,4R,5R,7S,9S,10S,11R,12S,13R)-7-[[2-(benzenesulfonamido)-1,1-dimethyl-ethyl]carbamoyloxy]-12-[(2R,4R,5S,6S)-4,5-dihydroxy-4,6-dimethyl-tetrahydropyran-2-yl]oxy-2-[(1S)-2-[(2R,3R,4R,5R,6R)-5-hydroxy-3,4-dimethoxy-6-methyl-tetrahydropyran-2-yl]oxy-1-methyl-ethyl]-10-[(2S,3R,4E,6R)-3-hydroxy-4-methoxyimino-6-methyl-tetrahydropyran (SEQ 009)

Same procedure as SEQ-569, from **S4**, was used while replacing 2-(2-Methyl-5-nitroimidazol-1-yl)ethylamine dihydrochloride with 1-amino-1,1-dimethy-2-benzenesulfonamido-ethane.

MS: method d

Retention time Tr (min) = 7.44; [M+Na]^+^ : m/z 1268

^1^H NMR Spectrum (500 MHz, δ in ppm, DMSO-d_6_): 0.79 (d, J=6.9 Hz, 3 H) 0.93 (m, 9 H) 1.10 (m, 30 H) 1.65 (s, 3 H) 1.76 (m, 5 H) 2.00 (m, 4 H) 2.14 (m, 3 H) 2.64 (m, 1 H) 2.71 (dd, J=12.3, 5.2 Hz, 1 H) 2.80 (t, J=9.0 Hz, 1 H) 2.89 (m, 1 H) 2.92 (dd, J=7.9, 2.5 Hz, 1 H) 2.98 (d, J=16.0 Hz, 1 H) 3.03 (m, 2 H) 3.29 (masked m, 1 H) 3.37 (s, 3 H) 3.40 (m, 1H) 3.45 (s, 3 H) 3.52 (m, 1 H) 3.60 (m, 1 H) 3.62 (s, 1 H) 3.69 (m, 1 H) 3.67 (t, J=2.5 Hz, 1 H) 3.76 (m, 1 H) 3.80 (s, 3 H) 3.85 (t, J=4.7 Hz, 1 H) 4.07 (s, 1 H) 4.36 (d, J=6.6 Hz, 1 H) 4.41 (d, J=8.0 Hz, 1 H) 4.43 (d, J=8.8 Hz, 1 H) 4.55 (m, 2 H) 4.86 (d, J=7.2 Hz, 1 H) 4.98 (d, J=1.9 Hz, 1 H) 5.14 (d, J=4.3 Hz, 1 H) 6.25 (s, 1 H) 7.52 (t, J=5.8 Hz, 1 H) 7.59 (d, J=7.7 Hz, 2 H) 7.63 (t, J=7.0 Hz, 1 H) 7.78 (d, J=7.2 Hz, 2 H)

Synthesis of (2R,3S,4R,5R,7S,9S,10S,11R,12S,13R)-7-[(benzylcarbamoyl)oxy]-2-(1-{[(2R,3R,4R,5R,6R)-5-hydroxy-3,4-dimethoxy-6-methyltetrahydro-2H-pyran-2-yl]oxy}propan-2-yl)-10-{[(2S,3R,6R)-3-hydroxy-4-(methoxyimino)-6-methyltetrahydro-2H-pyran-2-yl]oxy}-3,5,7,9,11,13-hexamethyl-6,14-dioxo-12-{[(2S,5R,7R)-2,4,5-trimethyl-1,4-oxazepan-7-yl]oxy}oxacyclotetradecan-4-yl 3-methylbutanoate (SEQ-372)

0.68 g of the compound SEQ-416 was placed in THF (7 ml). The solution obtained was cooled to 0°C. A solution of sodium metaperiodate in 7ml of water was then rapidly added. After 15 minutes at 0°C, the mixture was allowed to warm to rt and stirred for 6 hours. The precipitate formed was filtered and rinsed with 7 ml of THF. A 2N solution of methylamine in 1.21 ml of THF and then 139.04 μl of acetic acid were added. After stirring for 5 minutes at rt, 199.63 mg of NaBH_3_CN were added. The suspension obtained was stirred at rt for 20 hours. The precipitate formed was filtered off and rinsed with 50 ml of DCM. The filtrate was washed with 30 ml of saturated aqueous NaHCO_3_ solution and then with 30 ml of saturated aqueous NaCl solution. The aqueous phases were extracted with 50 ml of DCM. The organic phases were combined, dried over MgSO_4_, filtered and then evaporated to dryness under vacuum. The product obtained was purified by chromatography on a Merck cartridge (50 g of 15–40 μm silica), eluting with a 94/6 CHCl_3_/MeOH mixture, to afford 320 mg of the expected compound **SEQ-372**

MS: method b

Retention time Tr (min) = 1.31; [M-H+HCO_2_H]^-^: m/z 1166 (base peak).

^1^H NMR spectrum (500 MHz, in ppm, DMSO-d_6_ + CD_3_COOD): 0.81 (d, J=6.9 Hz, 3 H); 0.93 (d, J=6.8 Hz, 3 H); 0.95 to 1.02 (m, 9 H); 1.05 to 1.17 (m, 15 H); 1.24 (d, J=6.0 Hz, 3 H); 1.30 (d, J=6.6 Hz, 3 H); 1.74 (m, 7 H); 2.04 (m, 3 H); 2.18 (m, 5 H); 2.78 (s, 3 H); 2.79 (m, 1 H); 2.89 (d, J=14.8 Hz, 1 H); 2.93 (dd, J=2.5 and 8.0 Hz, 1 H); 3.03 (m, 2 H); 3.13 (m, 1 H); 3.35 (m, 2 H); 3.40 (s, 3 H); 3.45 (m, 1 H); 3.48 (s, 3 H); 3.51 (m, 2 H); 3.68 (m, 2 H); 3.82 (s, 3 H); 3.87 (m, 2 H); 3.94 (d, J=4.9 Hz, 1 H); 4.13 (d, J=5.7 Hz, 2 H); 4.45 (m, 2 H); 4.62 (m, 3 H); 5.12 (dd, J=4.5 and 8.4 Hz, 1 H); 7.28 (m, 6 H).

Synthesis of (2R,3S,4R,5R,7S,9S,10S,11R,12S,13R)-2-((S)-1-(((2R,3R,4R,5R,6R)-5-hydroxy-3,4-dimethoxy-6-methyltetrahydro-2H-pyran-2-yl)oxy)propan-2-yl)-10-(((2S,3R,6R)-3-hydroxy-4-(methoxyimino)-6-methyltetrahydro-2H-pyran-2-yl)oxy)-3,5,7,9,11,13-hexamethyl-7-(((2-methyl-1-((phenylsulfonamido)propan-2-yl)carbamoyl)oxy)-6,14-dioxo-12-(((2S,5R,7R)-2,4,5-trimethyl-1,4-oxazepan-7-yl)oxy)oxacyclotetradecan-4-yl 3-methylbutanoate (SEQ9).

Same procedure was used as SEQ-372, from SEQ009 instead of SEQ-416

MS: method b

Retention time Tr (min) = 1.15; [M+H]^+^: 1243

^1^H NMR spectrum (500MHz, in ppm, DMSO-d_6_): 0.77 (d, J=6.6 Hz, 3 H); 0.88 to 0.97 (m, 12 H); 1.0 to 1.15 (m, 27 H); 1.62 to 1.82 (m, 4 H); 1.67 (s, 3 H); 1.90 to 2.05 (m, 4 H); 2.09 to 2.20 (m, 4 H); 2.19 (s, 3 H); 2.39 (dd, J=9.7 and 13.9 Hz, 1 H); 2.59 (m, 1 H); 2.68 to 2.79 (m, 3 H); 2.83 to 2.93 (m, 3 H); 2.98 (broad q, J=6.6 Hz, 1 H); 3.03 (m, 1 H); 3.30 (m, 1 H); 3.37 (s, 3 H); 3.45 (s, 3 H); 3.52 (m, 1 H); 3.59 to 3.69 (m, 3 H); 3.76 (broad d, J=4.1 Hz, 1 H); 3.80 (s, 3 H); 3.83 (m, 1 H); 3.88 (t, J=4.8 Hz, 1 H); 4.28 (m, 1 H); 4.45 (d, J=8.0 Hz, 1 H); 4.59 (d, J=9.9 Hz, 1 H); 4.68 (m, 2 H); 4.87 (d, J=7.1 Hz, 1 H); 4.91 (dd, J=2.6 and 9.2 Hz, 1 H); 5.27 (d, J=4.8 Hz, 1 H); 6.22 (s, 1 H); 7.51 (broad t, J=6.6 Hz, 1 H); 7.58 (t, J=7.5 Hz, 2 H); 7.63 (t, J=7.5 Hz, 1 H); 7.78 (d, J=7.5 Hz, 2 H).

#### X-ray crystallography

*T. thermophilus* ribosomes were purified according to Jenner et al.^46^ Complexes with the 70S ribosome, tRNA^phe^ from *E. coli* and mRNA were formed before crystallization using sitting drop vapor diffusion at 22°C (Chryschem trays, Hampton research) with 5% PEGmmes 550, 5% PEG 20K, 200mM KSCN, 100mM Tris/HAc pH 7.0, 1.4mM Deoxy-Big Chaps. Unfortunately, co-crystallization with the antibiotics failed to show bound antibiotic in the resulting electron difference maps at concentrations that were consistent with optimal crystal growth (20mM). However, soaking macrolides into preformed crystals was successful in producing crystals which led to an interpretable electron density with bound inhibitors. Ribosome crystals were soaked with inhibitors for 24hrs in the cryo- mother liquor solution of 30% MPD, 5% PEGmmes 550, 5% PEG 20K, 200mM KSCN, 100mM Tris/HAc pH 7.0, 10mM MgOAc. Prior to synchrotron data collection at the PX06-I beamline, SLS, Switzerland, the crystals were cryocooled directly at the beamline in the stream of N_2_ (100K). Complete data sets were collected from 10 and 12 crystal (SEQ569 and SEQ977 respectively) and processed with the XDS package (Table S1). The structures were solved with molecular replacement and molecular refinement was carried out using PHENIX. The SEQ569 structure refined to an R_work_ of 21% and an R_free_ of 26% and nearly identical values were found for SEQ977 (Tables S1 and S2).

#### *Mtb* ribosome purification and in vitro translation assay

*Mtb* ribosomes were purified as previously described,^47^ except that methylated ribosomes were prepared from *Mtb* culture treated with 1 μg/ml of erythromycin. Briefly, cell lysates were clarified by centrifugation at 30,000 *g* for 1 h. The supernatant was pelleted in sucrose cushion buffer (20 mM HEPES pH 7.5, 1.1 M sucrose, 10 mM MgCl_2_, 0.5 M KCl, and 0.5 mM EDTA) at 185,511 *g* in a Beckman Type 45Ti rotor for 20 h. The pellet was resuspended in a buffer containing 20 mM Tris–HCl pH 7.5, 1.5 M (NH4)_2_SO_4_, 0.4 M KCl and 10 mM MgCl_2_. The suspension was then applied to a hydrophobic interaction column (Toyopearl Butyl-650S) and eluted with a reverse ionic strength gradient from 1.5 M to 0 M (NH_4_)_2_SO_4_ in a buffer containing 20 mM Tris–HCl pH 7.5, 0.4 M KCl, and 10 mM MgCl_2_. The eluted ribosome buffer was changed to the reassociation buffer (5 mM HEPES-NaOH, pH 7.5, 10 mM NH_4_Cl, 50 mM KCl, 10 mM MgCl_2_ and 6 mM β-mercaptoethanol) and protein was applied to a 10–40% linear sucrose gradient centrifuged in a Beckman SW28 rotor at 64,921 *g* for 19 h. The 70S fractions were concentrated to about A_260_ = 300 after removal of the sucrose.

The purified S30 fraction from *Mtb* lysate was prepared according to previous publication^48^ with modifications. Briefly, cell lysates were centrifuged twice at 30,000 *g* for 30 min at 4˚C. The supernatant was collected and 3 mL of pre-incubation buffer (0.3 M Tris-acetate, pH 8.2, 14.0 mM Mg(Ac)_2_, 12.0 mM ATP, 4.4 mM DTT, 0.04 mM 20 amino acids, 6.7 U/mL pyruvate kinase, 84.0 mM PEP) was added to every 10 mL of cell extract. The mixture was wrapped in aluminum foil and incubated at 37°C on a rotary shaker (120 rpm) for 80 min. Following the pre-incubation step, the S30 extract was dialyzed against four exchanges of 20 volumes of S30 buffer (10 mM tris-acetate pH 8.2, 14.0 mM Mg(Ac)_2_, 60 mM KAc, 1 mM DTT) for 45 min each. The dialyzed extract was centrifuged at 4000 *g* for 10 min at 4°C. The S100 faction was made from the S30 by centrifugation at 100,000 *g* for 2 hours at 4 ˚C on Beckman Type Ti50.2 rotor to remove ribosome.

The *Mtb* S100 extract (15 μL, containing translation factors such as initiation, elongation, termination, recycling factors and aminoacyl tRNA synthetase) was mixed with 5 μL 10X salt buffer (2 M potassium glutamate, 0.8 M ammonium acetate, and 0.16 M magnesium acetate), 0.5 mM each of the 20 amino acids, and 16.7 mM PEP. This mixture (41 μL) was combined with wild type or erythromycin - treated ribosome to a final concentration at 100 nM.

We next added 2 μL compound of each concentration (40 μM, 4 μM, 2 μM, 1 μM, 400 nM, 200 nM, 100 nM, 40 nM, 20 nM, 10 nM, 4 nM, 0) to the 41 μL mixture, and allowed it to incubate for 10 minutes at rt prior to adding master mix and mRNA. Translation was initiated by adding nanoluciferase mRNA (100 ng in 2 μL) and 5 μL 10X master mix (286 mM HEPES-KOH, pH7.5, 6 mM ATP, 4.3 mM GTP, 333 μM folinic acid, 853 μg/mL tRNA). The final volume was 50 μL. The reaction was allowed to proceed for one hour at 37°C at which time the reaction was terminated by the addition of 80 μM Chloramphenicol. The luminescent signal was detected by addition 20 μL of the nano-luciferase substrate Furimazine in S30 buffer.

#### Cryo-electron microscopy and data processing

SEQ-9-bound A2296-methylated *Mtb* 70S was prepared by incubating 800 nM methylated 70S with 16 μM SEQ-9 on ice for 30 min. 3 μl of the sample was applied to a glow-discharged Quantifoil R2/2 holey carbon grid (300 mesh), and vitrified using a Vitrobot Mark III (FEI company, The Netherlands) at 22°C with 100% relative humidity. Cryo-EM data were collected on a Titan Krios electron microscope (Thermo Fisher, USA) operating at 300 kV, at a nominal magnification of 130,000, which yields a pixel size of 1.06 Å/pixel. Image stacks were recorded on a Gatan K2 Summit (Gatan, Pleasanton CA, USA) direct detection camera in the electron counting mode. A total exposure time of 8.0 s with 0.2 s intervals and dose rate of ∼6.0 electrons/ Å^2^/s were used, resulting in 40 frames per image stack and accumulated total dose of ∼48 electrons per Å^2^.

Drift correction of collected image stacks was done by MotionCor2 with dose weighting.^44^ Aligned and summed image stacks were subjected to CTF estimation using gCTF.^45^ Images showing ice contamination and low resolution according to the estimation from gCTF were discarded, resulting in a total of 6,481 selected micrographs. Particle picking and reference-free 2D class average were done by Gautomatch (http://www.mrc-lmb.cam.ac.uk/kzhang/) and RELION-3.0,^49^ respectively. A total of 1,065,544 clean particles were selected and refined into one consensus map. Then 3D classification was used with the option ‘--skip_align’ to classify different states of ribosomes, and each state was processed according to the pipeline of RELION-3.0. The overall resolution was estimated according to the gold-standard Fourier shell correlation.^50^

SEQ-9-bound unmethylated *Mtb* 70S was prepared by incubating 700 nM unmethylated 70S with 16 μM SEQ-9 on ice for 30 min. 3 μl of the sample was applied to a glow-discharged Quantifoil R2/1 holey carbon grid (300 mesh) with 2nm carbon film, and vitrified using a Vitrobot Mark III (FEI company, The Netherlands) at 22°C with 100% relative humidity. Cryo-EM data were collected under a Titan Krios electron microscope (Thermo Fisher, USA) operating at 300 kV, at a nominal magnification of 165,000, which yields a pixel size of 0.83 Å/pixel. Image stacks were recorded on a Gatan K2 Summit (Gatan, Pleasanton CA, USA) direct detection camera in the electron counting mode. A total exposure time of 7.0 s with 0.2 s intervals and dose rate of ∼6.0 electrons/ Å^2^/s was used, resulting in 35 frames per image stack and accumulated total dose of ∼42 electrons per Å^2^.

Drift correction of collected image stacks was done by MotionCor2 with dose weighting.^44^ Aligned and summed image stacks were subjected to CTF estimation using Cryosparc Patch CTF estimation function.^39^ Images showing ice contamination and low resolution according to the estimation from Patch CTF were discarded, resulting in a total of 11,621 selected micrographs. Particle picking and reference-free 2D class average were done by Cryosparc.^39^ A total of 318,826 *Mtb* 70S particles were selected and refined into one consensus unmethylated *Mtb* 70S ribosome map at 2.8 Å (Figure S3A). To improve the resolution of the drug-binding site, we combined another 400,424 *Mtb* 50S particles in the same dataset from the same purification, yielding a total number of 719,250 particles of 70S and 50S. The drug-binding site was far away from the ribosomal subunit interface and was conserved in both the 70S and 50S, justifying the strategy to combine the 70S and 50S data to improve the resolution of the drug-binding site (Figure S3B). By applying a mask around the 50S subunit of the data mixing 70S and 50S, we obtained an unmethylated *Mtb* 50S ribosome map at 2.6 Å. The overall resolutions were estimated according to the gold-standard Fourier shell correlation (Relion 0.143 criterion).^50^ Final Fourier Shell Correlation plots for the density maps were generated using MATLAB R2021a.

#### Molecular modeling and refinement

The model of methylated *Mtb* 70S ribosome with SEQ-9, which was built starting with our previously published model from *Mtb* (PDB 5V93),^38^ was fitted into the calculated Cryo-EM map using rigid body fitting in Chimera.^40^ The docked model was then manually adjusted to refine into the Cryo-EM map using COOT.^41^ ERRASER^51^ was used to improve the RNA backbone geometry. Ligand restraints of SEQ-9 were generated by eLBOW.^52^ The SEQ-9 molecule was manually docked into the extra density around the PTC region, and refined using COOT. Iterative refinement using real space refinement in PHENIX^53^ and COOT was performed to improve the geometry and local fitting. Models of the unmethylated *Mtb* 70S/50S ribosomes with SEQ-9, were built in the same way as done for the methylated *Mtb* 70S ribosome with SEQ-9.

#### Microplate alamar Blue assay (MABA)

Minimum inhibitory concentrations (MICs) of test compounds for *Mtb* strains were determined as previously described^54^; For aerobic conditions where *Mtb* is in replicating conditions, the *Mtb* H37Rv strain was used in the MABA assay as follow. A *Mtb* PBS stock kept at -80°C was thawed and used to prepare a suspension at 10^5^ CFU/ml in Middlebrook 7H12 broth (Middlebrook 7H9 broth containing 0.1% w/v casitone, 5.6 μg/mL palmitic acid, 5 mg/mL bovine serum albumin, 4 μg/mL catalase), 100 μL of the suspension was distributed in 96-well microplates (black viewplates) containing 100 μL of 7H12 broth and 2μL of compounds dissolved and dilute in dimethylsulfoxide (DMSO), yielding a final testing volume of 200 μL with1% DMSO. Rifampin (RIF) and isoniazid (INH) were used as positive controls the plates were incubated at 37 °C and 5 % CO2 in plastic bags. On the sixth day of incubation, 50 μL of Alamar Blue (Promega) with 5% v/v Tween80 were added to each well; plates were then incubated at 37 °C for 16-24 h, and fluorescence of the wells was measured (λex = 530 nm, λem = 590 nm). In these experiments, the MIC was defined as the lowest concentration affecting an 80% reduction in fluorescence relative to the signal for the no drug control.

#### Microplate MIC assays with luciferase expressing *Mtb* (H37RvLuxAB)

*Mtb* strain H37Rv containing the inducible luciferase reporter plasmid pFCA-*luxAB* (LuxAB) was used to determine the MIC values of sequanamycins in the presence serum. 96-well white microplates were filled with 100μL of Middlebrook 7H12 medium supplemented with 25% of mouse or human serum and 2μL of different compounds concentrations or DMSO. A frozen LuxAB PBS stock was thawed and used to prepare a suspension at 10^5^ CFU/ml in the supplemented broth and 100 μL were distributed in each well. After 5 days incubation at 37 °C and 5 % CO2 in plastic bags, 50 μL of substrate (a 10% solution of *n*-decanal aldehyde in ethanol that was freshly diluted 10-fold in phosphate buffered saline were added in each well; plates were then sealed with a plastic film, and luminescence of each well was measured. The MIC was defined as the lowest concentration yielding an 80% reduction in luminescence relative to the signal for the no drug control.

#### Low-oxygen conditions for non-replicating bacteria

The low-oxygen recovery assay (LORA) was performed as described in Cho et al.^55^ The MIC was defined as the lowest concentration of the compound which reduces luminescence by 90% after 10 days of exposure to the compound under low oxygen followed by 28 h of aerobic recovery to allow metabolism wakeup compared to the untreated controls. The LORA minimal bactericidal concentration (MBC) value was the lowest concentrations of a given test compound which reduced the CFU by 99% after 10 days exposure relative to CFU at the start of the 10-day exposure. Comparative data for other antitubercular compounds targeting translation were described by can be found in Cho et al.^55^

#### MICs against *Mtb* H37Rv isogenic monoresistant strains and drug-resistant and drug-susceptible *Mtb* clinical isolates

MICs against *Mtb* H37Rv isogenic strains mono-resistant to rifampin (ATCC-35838), isoniazid (ATCC-35822), kanamycin (ATCC-35827), Cycloserin (ATCC-35826). The rMOX and rCAP strains were obtained by the Institute for Tuberculosis Research, University of Illinois at Chicago. MIC values against 6 clinical strains from different lineages and with different drug susceptibility profiles: KMA3899 Lineage 1, Indo-Oceanic (East African-Indian [EAI]); KMA4244 Lineage 2, East Asian (Beijing); KMA4439 Lineage 2, East Asian (Beijing); KMA1354 Lineage 3, East African-Asian (Central Asian [CAS]); KMA5282 Lineage 4, Euro-American (Haarlem, Latin-American Mediterranean [LAM], T, X); KMA5319 Lineage 4, Euro-American (Haarlem, LAM, T, X).

#### Intramacrophage assay

RAW264.7 murine macrophage-like cells (ATCC #TIB-71) were grown at 37 °C with 5% CO2 in Dulbecco’s modified Eagle medium supplemented with 10% fetal calf serum, 4 mM L-glutamine and 1 mM sodium pyruvate. Macrophages were seeded the day of the infection and separated into two flasks. One flask was used as the non-infected control. Green fluorescent protein (GFP)-expressing *Mtb* H37Rv were cultured for 6 days at 37 °C to rich a late log phase in Middlebrook 7H9 medium supplemented with 10% (v/v) oleic acid-albumin-dextrose catalase (OADC), 0.4% (v/v) glycerol and 0.05% (v/v) Tween 80 and used to inoculate the second flask containing the RAW264.7 cells at a multiplicity of infection of 2. After 2 h incubation at 37 °C with weak agitation, cells were washed twice with medium to remove extracellular bacteria. The infected cells were added, at a concentration of 10^5^ infected cells/well, to 96-wells plates (clear bottom black poly-D-lysine coated) containing the compounds to be tested. Uninfected and infected controls with 1% DMSO were included in the plates; Isoniazid and amikacin were used as positive and negative references, respectively. Plates were incubated for 4 days at 37 °C and 5% CO2. Infection was stopped by fixation with 4% paraformaldehyde for 30 min at rt followed by the infected macrophages staining with Cell Mask Blue (Invitrogen) diluted (1/5,000) in PBS/0.1% (v/v) Triton for 15 min at rt. Prior to image acquisition, wells were washed with PBS. Images were recorded on an automated fluorescent microscope Cellomics ArrayScan VTI HCS reader (Thermo). Each image was processed using the SpotDetector V3 BioApplication. The number of objects acquired per well was fixed (3,000 macrophages). Images contained two channels: one for the cell nuclei (Ch1; blue channel, HOECHST) and one for GFP-expressing *Mtb* (Ch2; green channel, FITC). For each well the intensity of green fluorescence per cell was recorded. This value was used to calculate the percent inhibition. The MIC_80_ was defined as the lowest drug concentration affecting signal inhibition of 80% relative to the signal for the high control.

#### Measurement of Sequanamycin intramacrophage accumulation

RAW264.7 cells were allowed to adhere in 96 wells plates for 4 hours before compounds were added at 10 μM. Intracellular concentrations were measured by LC-MS/MS after cellular lysis. For each point, up to 4 hours, the Intra/Extra cellular ratio was calculated. Points are the mean ± SD of four tests. Clarithromycin and Azithromycin were used as references.

#### Time-kill curve

An early logarithmic phase culture of *Mtb* strain H37Rv in Middlebrook 7H9 broth supplemented with 10% (v/v) OADC, 0.4% (v/v) glycerol and 0.05% (v/v) Tween 80 was used to prepare a suspension at a density of 5 × 10^5^ CFU/mL, as confirmed by quantitative plate counts. Compounds were solubilized and diluted in pure DMSO (for a final DMSO concentration of 1%). This fresh suspension was distributed in 125 mL Erlenmeyer flasks (25 mL/flask) and exposed to a range of compound concentrations or to a 1% DMSO for 14 days at 37 °C under shaking conditions. On days 2, 4, 7, 10 and 14, samples (500 μL) were taken for quantitative CFU plate counts. Samples were washed once by centrifugation at 14,000 × *g* for 10 min and resuspending the bacterial pellet in PBS to avoid possible carryover. The washed suspensions were serially diluted in PBS and plated onto antibiotic-free 7H10 agar plates supplemented with OADC. After 21 days of incubation at 37 °C and 5% CO2, CFU counts were determined. Log10 CFUs were plotted against time in days to obtain time-kill curves.

#### *Mtb* Ribosome purification and translation assay

The purified S30 fraction from *Mtb* was prepared according to previous publication (Methods Mol Biol. 2004; 267:169–182.) with modifications. Briefly, cell lysates were centrifuged twice at 30,000 *g* for 30 min at 4˚C. The supernatant was collected and 3 mL of pre-incubation buffer (0.3 M Tris-acetate, pH 8.2, 14.0 mM Mg(Ac)_2_, 12.0 mM ATP, 4.4 mM DTT, 0.04 mM 20 amino acids, 6.7 U/mL pyruvate kinase, 84.0 mM PEP) was added to every 10 mL of cell extract. The mixture was wrapped in aluminum foil and incubated at 37°C on a rotary shaker (120 rpm) for 80 min. Following the pre-incubation step, the S30 extract was dialyzed against four exchanges of 20 volumes of S30 buffer (10 mM tris-acetate pH 8.2, 14.0 mM Mg(Ac)_2_, 60 mM KAc, 1 mM DTT) for 45 min each. The dialyzed extract was centrifuged at 4000 *g* for 10 min at 4°C. Fraction S100 was made from S30 by centrifugation at 100,000 *g* for 2 hours at 4 ˚C on Beckman Type Ti50.2 rotor to remove ribosome.

*Mtb* ribosomes were purified as previously described,^47^ except that methylated ribosomes were prepared from *Mtb* culture with 1 μg/ml of erythromycin. Briefly, cell lysates were clarified by centrifugation at 30,000 *g* for 1 h. The supernatant was pelleted in sucrose cushion buffer (20 mM HEPES pH 7.5, 1.1 M sucrose, 10 mM MgCl_2_, 0.5 M KCl, and 0.5 mM EDTA) at 185,511 *g* in a Beckman Type 45Ti rotor for 20 h. The pellet was resuspended in a buffer containing 20 mM Tris–HCl pH 7.5, 1.5 M (NH4)_2_SO_4_, 0.4 M KCl and 10 mM MgCl_2_. The suspension was then applied to a hydrophobic interaction column (Toyopearl Butyl-650S) and eluted with a reverse ionic strength gradient from 1.5 M to 0 M (NH_4_)_2_SO_4_ in a buffer containing 20 mM Tris–HCl pH 7.5, 0.4 M KCl, and 10 mM MgCl_2_. The eluted ribosome peak was changed to reassociation buffer (5 mM HEPES-NaOH, pH 7.5, 10 mM NH_4_Cl, 50 mM KCl, 10 mM MgCl_2_ and 6 mM β-mercaptoethanol) then concentrated before applying to a 10–40% linear sucrose gradient centrifuged in a Beckman SW28 rotor at 64,921 *g* for 19 h. The 70S fractions were concentrated to about A260 = 300 after removal of the sucrose.

The *Mtb* S100 extract (15 μL, containing translation factors such as initiation, elongation, termination, recycling factors and aminoacyl tRNA synthetase) mixed with 10 μL 10X salt buffer (2 M potassium glutamate, 0.8 M ammonium acetate, and 0.16 M magnesium acetate), 0.5 mM each of the 20 amino acids, 16.7 mM PEP and 1% poly (ethylene glycol) 8000. This mixture (41 μL) was added to each well and mixed with wild type or erythromycin - treated ribosome to final concentration at 100 nM.

We next add 2 μL compound of each concentration (40 μM, 4 μM, 2 μM, 1 μM, 400 nM, 200 nM, 100 nM, 40 nM, 20 nM, 10 nM, 4 nM, 0) to the 41 μL mixture, and allow incubating for 10 minutes at rt prior to adding master mix and mRNA. The reaction was started by adding nanoluciferase mRNA (100 ng in 2 μL) and 5 μL 5X master mix (286 mM HEPES-KOH, pH7.5, 6 mM ATP, 4.3 mM GTP, 333 μM folinic acid, 853 μg/mL tRNA). The final volumn is 50 μL. The reaction was allowed to proceed for one hour at 37 degrees at which time the reaction was terminated by the addition of 80 μM Chloramphenicol. The luminescent signal was detected by addition 20 μL of the nano-luciferase substrate Furimazine in S30 buffer.

#### Generation of SEQ-9 resistant strains

10^9^ or 10^8^ CFU from an exponential *Mtb* H37Rv culture in Middlebrook 7H9 broth medium supplemented with 10% (v/v) oleic acid-albumin-dextrose-catalase (OADC), 0.4% (v/v) glycerol and 0.05% (v/v) Tween 80 were spread on 7H11 agar plates containing SEQ-9 at 4, 8 or 16 X MIC (7H11 agar powder, 0.5% glycerol,10% OADC) and allowed to growth at 37 °C and 5 % CO2 in plastic bags for 5 weeks. Among the SEQ-9 concentrations and the *Mtb* suspensions plates, 10 colonies were chosen and dispersed in 25 ml of 7H9 broth medium in 125 ml Erlenmeyer flask and incubated for 7 to 13 days with shaking until DO_600_ ∼0.8. Cultures were then centrifuged, and cells were frozen at -80°C in 15% Glycerol for long conservation (primary stock). PBS stocks were prepared from this primary stock to evaluate the resistance profile to various molecules in the MABA test previously described. Stability of the mutation was assessed in the MABA test on cultures obtained after 3 consecutive passages without SEQ-9. DNA from each mutant was extracted using MaxWell RSC Instrument with the Maxwell RSC Cultured Cells DNA Kit (Promega) according to the manufacturer’s instruction. DNA concentration was measured with nanodrop (Thermo Scientific) and sequenced.

#### Frequency of resistance

Four replicate cultures of *M. tuberculosis* H37Rv were prepared in 50-ml flasks, each containing 10 ml Middlebrook 7H9 broth plus glycerol, Casitone and oleic acid-albumin- dextrose-catalase (OADC) supplement at an initial density of *Mtb* of <10^4^ CFU/ml and monitored for the presence of preexisting resistant bacteria by plating 100μl aliquots on medium containing the test compound at 4X MIC. Cultures were then incubated for 2 to 3 weeks with shaking until an OD570 of >0.9. From each of four cultures, 100μl aliquots of undiluted and 1:10 diluted suspensions (approximately 1 X 10^9^ CFU/ml and 1 X 10^8^ CFU/ml, respectively) were plated on 10 plates of 7H11 agar with and without 4X MIC of SEQ9. Individual mutation frequencies were calculated for each of the four cultures, and the median value was selected as representative.

#### Overexpression of *Erm37*

*M. bovis BCG Pasteur* (IP1173P2) and Moreau (TMC1013 (BCG Brazilian), ATCC35736) strains were cultivated in Middlebrook 7H9 broth supplemented with 10% (v/v) OADC, 0.4% (v/v) glycerol and 0.05% (v/v) Tween 80. Minimum inhibitory concentrations (MICs) of test compounds for *M. bovis* strains were determined as described above except that *M. bovis* was cultivated in Middlebrook 7H9 broth supplemented with 10% (v/v) OADC, 0.4% (v/v) glycerol and 0.05% (v/v) Tween 80 (Middlebrook 7H9 broth supplemented with 0.2% glycerol, 0.05% Tween 80; 0. 6% v/v oleic acid, 2 mg/mL dextrose, 5 mg/mL bovine serum albumin, 3 μg/mL catalase). The *erm37* ORF was PCR amplified using oligonucleotides 1: CTCGATCATATGGTGTCCGCCCTCGGACGGTC and 2: CATACTAAGCTTTTACC GCCCCTGCCAGTCAC and genomic DNA from *M. bovis* Moreau strain. The Rv0560c promoter (1021 bp upstream the ATG start codon) was PCR amplified using oligonucleotides 3: ATCGTTGACGTCGCGG CCGCATCGTGGGTTGCGGATGAGC and 4: ATCGCTGAATTCGTGTTCATATATATCAACGGC and genomic DNA from *M. bovis BCG Pasteur* as a matrix. The *erm37* ORF was cloned in between the NdeI and HindIII sites under the control of the Rv0560c promoter inserted between the NotI and NdeI sites in a shuttle vector (a kind gift from E. Liauzun) containing an origin of replication for Mycobateria (OriM), an origin of replication for *E. coli* (OriC) and the kanamycin resistance cassette. Another promoter, MMAR5083, corresponding to the gene upstream of lpqE in *M. marinum*, was inserted in place of Rv0560c in the same vector and led to the same results.

The entire *erm37* gene, including its own promoter, was PCR amplified using oligonucleotides 2 and 5: ATCGTCGCGGCCGCGATATCGCCTCATTGGC, as described above, and cloned in between the NotI and HindIII sites in the same vector. All the molecular biology cloning experiments were performed according to the manufacturer recommendations. We used *E. coli* Top10 cells (Invitrogen) for cloning experiments. We prepared and transformed *M. bovis* BCG by electroporation as described,^56^ using 200μl aliquots stored in 10% glycerol at -80°C, except that bacteria were recovered in 1ml SOC medium (Sigma) for 6h at 37°C. Then, *M. bovis* BCG transformed bacteria were cultivated in the presence of 20μg/ml kanamycin.

#### RNA extraction and erm37 mRNA quantification

A mid log phase *Mtb* H37Rv culture was incubated overnight in Middlebrook 7H9 broth (supplemented with + 10 % OADC + 0.05% Tween80 + 0.4% glycérol) with subinhibitory concentrations of clarithromycin or SEQ-9 (0.125μg/ml and 0.25μg/ml). MICs were then determined for each culture using a microplate Alamar blue assay as described above. For RNA extraction, cells were lysed in Thioglycerol with fast prep instrument (MP Biomedicals) and total RNA was extracted using MaxWell RSC Instrument with the Maxwell RSC simply RNA Cells kit (Promega) according to the manufacturer’s protocol. RNA concentration was measured with nanodrop (Thermo Scientific) and RNA quality was evaluated by Bioanalyzer (Agilent). Reverse transcription of total mRNA was performed with 500 ng total RNA using SuperScript™ IV VILO (Applied Biosystems) and quantified using 7500 Real-Time PCR system (Applied Biosystems) The data was analyzed using ThermoFisher ConnectTM online application (ThermoFisher). The mRNA content was normalized to 23S expression. GraphPad Prism 8 was used for statistical analysis using Student’s t-test (dCT). Probes used for mRNA detection were erm37 (F: AGCGATTCCCTGGCATTACC; R: GCGGGTTCGCCACAAC; FAM, ACGCCGCCTCGATCC); for 23S (F: GCGCCCGTGACGAATC, R: ACGCAGCCCCAGAACTC, FAM: TAACCACCCAAAACCG).

#### Metabolic stability in human liver microsomes

The metabolic stability of test compounds was determined as described.^57^^,^^58^ Incubation and experimental conditions with human hepatic microsomal fractions (purchased from BD Gentest as a pooled batch from 6 donors) were as follows: microsomal proteins, 1 mg/mL; bovine serum albumin, 1 mg/mL; substrate, 5 μM; incubation duration, 20 min; cytochrome P-450 monooxygenases (CYPs) and flavin containing monooxygenases (FMOs) cofactor, 1 mM NADPH. Enzyme activity was stopped with 1 volume of acetonitrile. Ketoconazole at a final concentration of 1.5 μM (100-fold above its Ki for CYP3A4) was used for the specific and potent inhibition of enzyme reactions catalyzed by CYP3A4. For each test compound and for each microsomal preparation, three incubations were prepared: absolute reference in buffer (without enzyme material, i.e., microsomes); incubation without NADPH cofactor (with microsomal fractions); and incubation with NADPH (with microsomal fractions). For most compounds, biotransformation, as observed in hepatic microsomal fractions in the presence of the NADPH cofactor, consists of oxidative reactions catalyzed by either CYPs or FMOs. In these conditions, the percentage of total metabolism, which corresponds to oxidative metabolism, was determined as follows: [% total metabolism] ≈ [% oxidative metabolism] = [unchanged compound (UC) peak area − NADPH UC peak area + NADPH] × 100%, where NADPH corresponds to the enzyme cofactor for oxidation reactions catalyzed by either CYP or FMO.

#### Inhibition studies using human liver microsomes

Incubations were performed with 0.1 mg/mL human liver microsomes (purchased from BD Gentest as a pooled batch from 150 donors) in a final incubation volume of 0.25 mL. The incubation medium contained 0.05 M phosphate buffer (pH 7.4) containing an NADPH regenerating system (including 1.3 mM NADP, 3.3 mM glucose 6-phosphate,3.3 mM MgCl2, and 1.0 U/mL glucose 6-phosphate dehydrogenase). Probe CYP3A4 substrates (midazolam at 3 μM and testosterone at 50 μM), were incubated for 10 and 30 min, respectively, with increasing concentrations of GM, MGM or CGM (concentration range: 1 - 30 μM). The reaction was stopped by adding acetonitrile to precipitate the proteins. The incubation mixtures were then centrifuged for 5 min at 10,000 × *g*, and an aliquot of the supernatant was analyzed using high-performance liquid chromatography coupled to mass spectrometry for the assessment of 1’-hydroxymidazolam and 6β-hydroxytestosterone metabolite formation.

#### Pharmacokinetic studies in mice

The systemic and pulmonary exposure to sequanamycins was assessed following a single intravenous (1.5 or 3 mg/kg expressed as active compound) bolus or a single oral administration (10, 30, 100, 300 mg/kg expressed as active compound) to male Swiss mice (Janvier Laboratories, Saint Isle, France). Mice were fed ad libitum. For the oral route, the test compound was administered in either 6% Solutol/ PBS, or NMP / Solutol HS15 / PBS pH 7.4 (10%/5%/ 85%) 0.6% Methylcellullose/ Tween 80 (99.5/0.5; v/v) and for SEQ-9 PEG200/Tween/80/Citrate buffer 100mM– pH 4 (70/5/25) or Cremophor RH40 / Capryol 90 / Mygliol 812N (10%/20%/70%; w/w/w) diluted ½ in water corresponding to vehicle used for *Mtb* mouse models. Samples were collected at various times; three mice were sacrificed per group per time point. Blood samples were collected by decapitation at each sampling time in tubes containing lithium heparin as anticoagulant. After centrifugation (10,000 × *g* for 5 min at 20 °C), plasma samples were transferred into 1.5 mL tubes and stored at -20 °C until analysis. In some cases, lungs were collected at each sampling time, weighed and frozen at -20 °C until analysis. All animal pharmacokinetic experiments were performed in an animal biosafety level-1 facility. All animals were cared for in strict accordance with the guidelines of and the protocols approved by the Sanofi Ethical Committee for Animal Laboratory (Protocol T42141/010). Analyses were performed on an Agilent 1100 system equipped with a binary pump, a heater, an Agilent 1100 well plate autosampler and a Thermo TSQ Quantum Ultra EMR MS detector (ionization mode: ESI+) run with Excalibur version 1.4 software. Chromatography was performed on a Phenomenex Luna C5 (50 × 2 mm, 5μm) column heated at 35 °C at a flow of 0.20 mL/min with an injection volume of 20 μL. Mobile phase A: NH4OAc (0.15 g)/HCOOH (2 mL)/HPLC grade water up to 1000 mL; B: NH4OAc (0.15 g)/HCOOH (2 mL)/MeOH (200 mL)/MeCN up to 1000 mL. Gradient mode: t0: 50% B, t0.1 min: 50% B, t0.5 min: 90% B, t5 min: 90% B, t5.5 min: 50%B, t12 min: 50% B. Quality control and calibration samples were prepared daily by spiking mouse plasma or tissue homogenates (lung and liver) with working solutions prepared from independent weightings. The calibration curve was calculated from levels at 0.5, 1, 2.5, 5, 10, 25, 50, 100, 200, 500, 1000 and 2000 ng/mL (n = 3 per concentration). Quadratic regressions were not forced through the origin and weighted by 1/x. The limits of quantification were estimated to be 0.5 ng/mL for the plasma and to 1 ng/g for the lung and liver, one gram of tissue being mixed with 1 mL of water for homogenization. The pharmacokinetic parameters were calculated from the arithmetic mean of the plasma concentrations or the tissue concentrations using the software WinNonLin 5.2, non-compartmental models 200 and 201.

Iv:

SEQ503/3/ Solution in 6 % Solutol in PBS
SEQ416/3/ 6% Solutol/ PBS
SEQ372/3/ NMP / Solutol HS15 / PBS pH 7.4 (5%/5%/90%)
SEQ9/ 1.5/ NMP /Solutol HS15/ PBS pH 7.4 (10%/5%/ 85 %)
Po
SEQ503/ 10/ Solution in 10 % Solutol in PBS
SEQ416/30/ 10% Solutol/ PBS
SEQ372/30/ 0.6% Methylcellullose/ Tween 80(99.5/0.5; v/v)
SEQ9 /10/ 0.6% Methylcellullose/ Tween 80 (99.5/0.5; v/v) /PEG200/Tween/80/Citrate buffer 100mM– pH 4 (70/5/25) / Cremophor RH40 / Capryol 90 / Mygliol 812N (10%/20%/70%; w/w/w) diluted ½ in water (^∗^)

#### Mouse model of tuberculosis

For all in vivo models, Isoniazid (INH) and rifampicin (RIF) were prepared as a suspension of 0.6% methylcellulose with 0.5% Tween-80 (MCT), linezolid in PEG200 / ME (0.5% ME) (5% / 95%) and SEQ-9 was prepared either as a suspension in 0.6% methylcellulose with 0.5% Tween-80 (MCT) or as an emulsion in Cremophor RH40 / Capryol 90 /Mygliol 812N (10%/20%/70%;w/w/w) diluted ½ in water (Lip/2 ).

For the acute and highly acute models of Tuberculosis, the mouse treatment procedures were approved by the Animal Care and Use Committee of Sanofi. Female BALB/c mice aged 5 to 7 weeks were purchased from Janvier Laboratories, France. Mice were housed at 5 animals per cage with sterile bedding in HEPA–filtered racks (Tecniplast, France) in certified animal biosafety level 3 (ABSL-3) laboratories. Temperatures were maintained at 65-75°F (∼18-23 °C) with 40-60% humidity. Specific pathogen-free status was verified by testing sentinel mice housed within the same rack. Mice had ad libitum access to water and food pellets. Mice were rested 1 week before infection, then exposed to H37Rv *Mtb*, from a freshly grown culture of *Mtb*, by the intranasal route. Five mice were sacrificed the following day to determine the number of CFU implanted in the lungs and five mice were also sacrificed the day of treatment initiation to establish the pre-treatment lung CFU burden. All treatment was administered daily (5 days per week) by oral gavage. During the study, the mice were observed and weighed daily. Lungs were aseptically removed and homogenized in PBS/glycerol.

The number of viable organisms was determined by plating serial dilutions in PBS on Middlebrook 7H11 agar supplemented with OADC 10% and glycerol 0.5%. Plates were incubated at 37 °C for 3 weeks. Statistical analysis was performed by a one-way analysis of variance (ANOVA), followed by a multiple comparison analysis of variance by Dunnett’s test. Differences were considered significant at the 95% level of confidence. Significance was evaluated versus the control group: ^∗^p<0.05 ^∗∗^p<0.01, ^∗∗∗^p<0.001.

For mice chronic model, mice were inoculated via aerosol by *Mtb* Erdman bacteria. Compounds were administered daily (5 days per week) by oral gavage for 4 weeks and dosing was initiated 4 weeks post-infection. The protocol results in the deposition of approximately 50 to 100 bacilli into the lungs, and the course of infection was then followed by plating homogenates of the lungs on 7H11 agar and determining CFU. Controls consisted of mice treated with the vehicle only. Data represent the mean lung log_10_ CFU counts (five mice per group). Mice were sacrificed 3 days after the final dose to minimize carryover from the lung homogenates to the plating medium. For statistical analysis, the CFU counts were converted to logarithms values (Log_10_ CFU), which were then evaluated by a non-parametric statistical test, the Kruskal Wallis test.

For combination studies, all mice were purchased from Charles River (Wilmington, MA, USA). All infection experiments were performed in an animal biosafety level-3 facility at the Johns Hopkins University School of Medicine; all animals were cared for in strict accordance with the guidelines of and the protocols approved by the University’s Animal Care and Use Committee (Protocol MO10M59). Assessment of SEQ-9 in combination with other anti-TB drugs during the intensive and continuation phases of TB chemotherapy were performed in an acute mouse model of TB (using female BALB/c mice 6-8 weeks old) where mice were infected with 3-3.5 log_10_ CFU, and treatment was initiated two weeks after infection. Groups of 5 mice per group were sacrificed after 4 and 8 weeks of treatment to determine lung CFU counts.

Doses tested were: SEQ-9 300 mg/kg/day; Bedaquiline (B) 25 mg/kg/day; Pretomanid (Pa) 100 mg/kg/day; linezolid (L) 100 mg/kg/day; Pyrazinamide (Z) 150 mg/kg/day. Linezolid and Pyrazinamide were formulated in distilled water each week. Pretomanid was prepared in a cyclodextrin micelle formulation (CM-2) monthly, and diluted each week. Bedaquiline was prepared in an acidified HPCD solution every 2 weeks. SEQ9 was prepared weekly in PEG200/Tween 80/Citrate buffer 100mM pH4 (70/5/25). SEQ9 was administered 3-4 hr after other drugs. Each drug was administered once daily by gavage for 5 days/week.

### Quantification and statistical analysis

Particle picking and reference-free 2D class average were done by Gautomatch (http://www.mrc-lmb.cam.ac.uk/kzhang/) and RELION-3.0.^49^ The overall resolutions were estimated according to the gold-standard Fourier shell correlation (Relion 0.143 criterion).^50^ The mRNA content was normalized to 23S expression. GraphPad Prism 8 was used for statistical analysis using Student’s t-test (dCT). Probes used for mRNA detection were erm37 (F: AGCGATTCCCTGGCATTACC; R: GCGGGTTCGCCACAAC; FAM, ACGCCGCCTCGATCC); for 23S (F: GCGCCCGTGACGAATC, R: ACGCAGCCCCAGAACTC, FAM: TAACCACCCAAAACCG).

## Data Availability

• Atomic coordinates of Methylated Mtb Ribosome 70S with Seq-9, and Unmethylated Mtb Ribosome 50S with Seq-9 have been deposited in the PDB (http://www.rcsb.org) and are publicly available as of the date of publication under the accession codes PDB: 7KGB and PDB: 7SFR, respectively. The corresponding EM maps of Methylated Mtb Ribosome 70S with Seq-9, and Unmethylated Mtb Ribosome 50S with Seq-9 have been deposited in the Electron Microscopy DataBank (https://www.ebi.ac.uk/pdbe/emdb/), under the accession codes EMDB: EMD-22865 and EMD-25100, respectively. These accession numbers are also listed in the key resources table. All other data are available from the corresponding authors upon reasonable request. • This paper does not report original code. • Any additional information required to reanalyze the data reported in this paper is available from the lead contact upon request.
