# Supplementary material for: Discovery of natural-product-derived sequanamycins as potent oral anti-tuberculosis agents
Source: Cell. 2023 Mar 2;186(5):1013–1025.e24. doi: 10.1016/j.cell.2023.01.043 (PMC9994261; doi:10.1016/j.cell.2023.01.043)
Supplement: Document S1. Tables S1–S8 [file mmc1.pdf]

Cell, Volume *186*

Supplemental information

Discovery of natural-product-derived sequanamycins as potent oral anti-tuberculosis agents

Jidong Zhang, Christine Lair, Christine Roubert, Kwame Amaning, María Belén Barrio, Yannick Benedetti, Zhicheng Cui, Zhongliang Xing, Xiaojun Li, Scott G. Franzblau, Nicolas Baurin, Florence Bordon-Pallier, Cathy Cantalloube, Stephanie Sans, Sandra Silve, Isabelle Blanc, Laurent Fraisse, Alexey Rak, Lasse B. Jenner, Gulnara Yusupova, Marat Yusupov, Junjie Zhang, Takushi Kaneko, T.J. Yang, Nader Fotouhi, Eric Nuermberger, Sandeep Tyagi, Fabrice Betoudji, Anna Upton, James C. Sacchettini, and Sophie Lagrange

Table S1. X-ray Data Collection Statistics for SEQ-569 and SEQ-977 bound to the *T. thermophilus* ribosome, Related to Figure 1

Data statistics:	SEQ569	SEQ977
# Crystals	10	16
Resolution	300-3.2Å (3.3-3.2)	300-3.4Å (3.5-3.4)
Completeness	100.0% (100.0%)	100.0% (100.0%)
I/dI	9.6 (2.1)	9.7 (2.1)
R_{mrgd}	19.6% (83.4%)	18.2% (76.9%)
Obs #	18,957,688	24,148,767
Unique #	966,671	787,354
Redundancy	19.6	30.7

*numbers in parenthesis are for the outer resolution shell

Table S2. X-ray Refinement Statistics for SEQ-569 and SEQ-977 bound to *T. thermophilus* ribosome crystal structure, Related to Figure 1

Refinement statistics:	SEQ569	SEQ977
Resolution	300-3.2Å (3.3-3.2)	300-3.4Å (3.5-3.4)
Rwork / Rfree	0.21 / 0.26 (0.28 / 0.33)	0.21 / 0.26 (0.27 / 0.32)
# atoms	306,542 (2 molecules)<br>~18,000 nucleotides<br>~20,000 amino acids	305,650 (2 molecules)<br>~18,000 nucleotides<br>~20,000 amino acids
# ions (Os/Mg)	1,632	1,384
Rmsd bonds (Å)/ angles (degrees)	0.011 / 1.353	0.68 / 1.366

*numbers in parenthesis are for the outer resolution shell

Table S3. Inhibition concentration for macrolides determined in a *Mtb* translation assay using methylated and non-methylated ribosome, Related to STAR Methods section – Mtb ribosome purification and in vitro translation assay

	IC_{50} ribosome (nM)	IC_{50} methylated ribosome (nM)
Erythromycin	671	140000
Clarithromycin	354	88100
Azithromycin	2910	>100000
Tylosin	89.2	377
Clindamycin	80	42300
SEQ-9	75	64

Table S4. Growth inhibition measurement (MIC_{80}, µM) in inducible erm37 overexpression M. bovis BCG Pasteur strain suggests that expression of erm37 confers resistance to classical macrolides, but not to sequanamycines, Related to STAR Methods section – Microplate Alamar Blue Assay (MABA)

	Clarithromycin	SEQ-372	SEQ-9	Erythromycin	Linezolid	Isoniazid
Control	0.07	0.28	0.32	0.16	1.33	0.34
pRv0560c:erm37	32.0	0.29	0.56	33.0	1.43	0.40
pLpqE-erm37	33.0	0.25	ND	ND	3.28	ND
pErm37-erm37	0.76	0.34	ND	ND	2.89	ND

M. bovis BCG Pasteur was transformed with an empty plasmid (control) or a plasmid allowing expression of erm37 under the control of the different promoters; Rv0560c (pRv0560c:erm37) or MMAR5083, corresponding to the gene upstream of lpqE in *M. marinum*, (pLpqE-erm37) .The entire erm37 gene, including its own promoter (pErm37-erm37) was also cloned and transformed. MIC_{80} (µM) were assessed for each condition and results were the average of three independent experiments.

Table S5: Overnight pre-incubation with clarithromycin or SEQ-9 induces erm37 mRNA level and increase clarithromycin resistance, Related to STAR Methods section – Overexpression of Erm37 & RNA extraction and erm37 RNA quantification

		MIC_{80} Clarithromycin	MIC_{80} linezolid	MIC_{80} SEQ-9	erm37 mRNA fold change
**Without induction**		2	1	1	1
**Clarithromycin**	0.125µg/ml	8	1	1	-
	0.25µg/ml	8	1	1	57 *
**SEQ-9**	0.125µg/ml	4	1	1	3
	0.25µg/ml	8	1	1	12 *

H37Rv culture was incubated overnight in Middlebrook 7H9 broth with sub-inhibitory concentrations of clarithromycin or SEQ-9. MIC_{80} (µM) were determined for each condition. The level of erm37 mRNA was measured by RT-qPCR and results are the mean of the fold induction compared to untreated controls. Each sample was normalized to the level of 23S ribosomal RNA. Results are the average of three independent experiments (*p>0.05, Student's t-test).

Table S6. Growth inhibition measurement (MIC_{80}, µM) in SEQ-9 resistant strains exhibiting mutations on ribosomal 23S gene, Related to STAR Methods section – Generation of SEQ-9 resistant strains

23S mutation	H37Rv WT	Mutant A2296G	Mutant Del 1848G	Mutant Del 872G	Mutant Del 874T
SEQ-372	0.50	7.3	-	12.0	2,92
SEQ-416	0.26	2.6	-	10.1	1,25
SEQ-9	0.58	13.2	8.7	12.5	9,91
BDQ	0.044	0.042	-	0.036	0.042
Linezolid	0.92	0.95	1.14	0.80	0.79
Rifampicin	0.098	0.043	0.029	0.019	0.022
Clarithromycin	1.19	NA @ 30µM	21.4	55% @ 30µM	70% @ 30µM

H37Rv resistant clones were selected on agar plates with SEQ-9 added. Ten clones were isolated for whole genome sequencing and mapped to the *M. tuberculosis* H37Rv genome sequence. Mutations in the table were annotated with the *Mtb* rrl sequence. Resistance evaluation, MICs (µM) were assessed for Sequanamycins, Clarithromycin and other TB drugs (Results/ MICs (µM)) using a microplate Alamar blue assay (see Methods). Results were the average of three independent experiments.

Table S7. Mean pharmacokinetic parameters for SEQ-9 in mice following p.o single dose, Related to STAR Methods section –Pharmacokinetic studies in mice

			Plasma			Lung		
**Formulation**	**Route**	**Doses** mg/kg	**C_{max}** µg/ml	**$t^{1/2}$** hours	**AUC_{0-24}** µg.h/ml	**C_{max}** µg/ml	**$t^{1/2}$** hours	**AUC_{0-24}** µg.h/ml
PEG200/Tween80/Citrate Buffer 100mM pH4 (70/5/25)	po	10	0.14	3.0	1.1	0.79	4.5	7.8
	po	100	1.39	4.4	11	32.9	6.5	190
	po	300	3.11	nd	21	252	11	840
Lipidic formulation: CremophorRH40/Capryol90 / Mygliol812N (10/20/70) diluted in ½ water	po	100	2.13	4.2	22	31.9	4.5	440

Cmax, maximum concentration; AUC, area under the concentration curve; t½, half-life; nd, not determined. Plasma samples were collected from three mice per time point and SEQ-9 concentrations were determined by LC-MS/MS.

Table S8: Lung CFU counts from the combination experiment, Related to Figure 6

		Time point and mean ± SD lung CFU counts			
**Regimen**	**N**	**Day -13**	**Day 0**	**Week 4**	**Week8**
**Untreated**	5	3.69±0.02		ND	
	5		6.92±0.05		
**X 300**	4			6.83±0.17	
**B 25**	4			2.96±0.19	
**Pa 100**	4			5.83±0.12	
**L 100**	4			6.86±0.08	
**Z 150**	4			5.99±0.19	
**BPa**	4/5			3.65±0.11	1.69±0.23
**BX**	5/4			2.07±0.30	0.85±0.68
**PaX**	4/5			4.93±0.16	2.07±0.59
**LX**	4/5			6.36±0.04	5.44±0.07
**ZX**	4/4			5.24±0.26	4.24±0.19
**BPaX**	5/3			3.19±0.40	0.20±0.17

Drug doses: SEQ-9 300 mg/kg/day; Bedaquiline (B) 25 mg/kg/day; Pretomanid (Pa) 100 mg/kg/day; linezolid (L) 100 mg/kg/day; Pyrazinamide (Z) 150 mg/kg/day. N* indicates the number of mice per group.
